# Charge midwives’ awareness of and their role in promoting respectful maternity care at a tertiary health facility in Ghana: A qualitative study

**DOI:** 10.1371/journal.pone.0284326

**Published:** 2023-05-15

**Authors:** Veronica Millicent Dzomeku, Adwoa Bemah Boamah Mensah, Emmanuel Kweku Nakua, Pascal Agbadi, Joshua Okyere, Richard Gyan Aboagye, Peter Donkor, Jody R. Lori

**Affiliations:** 1 Department of Nursing, College of Health Sciences, Kwame Nkrumah University of Science and Technology, Kumasi, Ghana; 2 Department of Epidemiology and Biostatistics, School of Public Health, Kwame Nkrumah University of Science and Technology, Kumasi, Ghana; 3 Department of Sociology and Social Policy, Lingnan University, Tuen Mun, Hong Kong; 4 Department of Family and Community Health, Fred N. Binka School of Public Health, University of Health and Allied Sciences, Hohoe, Ghana; 5 Department of Surgery, School of Medical Sciences, Kwame Nkrumah University of Science and Technology, Kumasi, Ghana; 6 Komfo Anokye Teaching Hospital, Kumasi, Ghana; Waikato Institute of Technology, NEW ZEALAND

## Abstract

**Background:**

Evidence suggests that the implementation of respectful maternity care (RMC) interventions is one of the surest and most effective means of minimising mistreatment during intrapartum care services. However, to ensure the successful implementation of RMC interventions, maternity care providers would have to be aware of RMC, its relevance, and their role in promoting RMC. We explored the awareness and role of charge midwives in promoting RMC at a tertiary health facility in Ghana.

**Methods:**

The study adopted an exploratory descriptive qualitative study design. We conducted nine interviews with charge midwives. All audio data were transcribed verbatim and exported to NVivo-12 for data management and analyses.

**Results:**

The study revealed that charge midwives are aware of RMC. Specifically, ward-in-charges perceived RMC as consisting of showing dignity, respect, and privacy, as well as providing women-centred care. Our findings showed that the roles of ward-in-charges included training midwives on RMC and leading by example, showing empathy and establishing friendly relationships with clients, receiving and addressing clients’ concerns, and monitoring and supervising midwives.

**Conclusion:**

We conclude that charge midwives have an important role to play in promoting RMC, which transcends simply providing maternity care. Policymakers and healthcare managers should ensure that charge midwives receive adequate and regular training on RMC. This training should be comprehensive, covering aspects such as effective communication, privacy and confidentiality, informed consent, and women-centred care. The study also underscores a need for policymakers and health facility managers to prioritise the provision of resources and support for the implementation of RMC policies and guidelines in all healthcare facilities. This will ensure that healthcare providers have the necessary tools and resources to provide RMC to clients.

## Introduction

Between September 2000 and the end of 2015, the millennium development goals (MDGs) on maternal health focused primarily on reducing maternal mortality through concerted efforts to increase skilled birth attendance and institutional birth delivery [1]. This strategy resulted in a significant decline in the global maternal mortality ratio from 385 maternal deaths per 100,000 live births in 1990 to 216 in 2015, thus indicating a 44 percent decline [2,3]. Yet, low-and-middle-income countries (LMICs) such as Ghana continue to have a high maternal mortality ratio (MMR) [4]. For example, a report from the 2017 Ghana maternal health survey shows that Ghana has an MMR of 380 deaths per 100,000 live births [5]. Moreover, the perinatal mortality rate in Ghana was estimated at 21.7 per 1000 births in 2019 [6].

To ensure further and sustained reduction in maternal mortality worldwide, the United Nations together with its member countries designed and implemented the 17 Sustainable Development Goals (SDGs) 3, which target 3.1 envisions to reduce maternal mortality ratio to less than 70 deaths per 100,000 live births [3]. Hence, in many countries where MMR is high, especially in the case of sub-Saharan Africa, several interventions and programmes geared toward encouraging women to have institutional birth delivery was initiated [7]. For instance, in Ghana, the government initiated the free maternal healthcare policy which aimed to increase institutional delivery [8]. Nevertheless, it is important to note that this promotion of institutional birth delivery is fraught with some challenges as many women report experiencing obstetric violence and abuse at the hands of maternity care providers [9]. Hence, raising concerns about promoting respectful maternity care during the continuum of pregnancy.

Respectful maternity care (RMC) refers to the “care organized for and provided to all women in a manner that maintains their dignity, privacy, and confidentiality, ensures freedom from harm and mistreatment, and enables informed choice and continuous support during labour and childbirth” [10]. Thus, having access to dignified maternity care through RMC is every woman’s right [11]. It is important to note that denying RMC to pregnant women could manifest through physical, verbal, or psychological abuse. Evidence shows that abuses suffered by pregnant women at the hands of maternity care providers is partly responsible for several mortalities and disabilities during pregnancy and childbirth [12].

There is a plethora of evidence suggesting that the implementation of RMC interventions is one of the surest and most effective means of minimising mistreatment during intrapartum care services [13–15]. However, to ensure the successful implementation of RMC interventions, maternity care providers would have to be aware of RMC, its relevance, and their role in promoting RMC. Traditionally, the maternity unit of a health facility in Ghana is made up of a doctor, physician, nurses, and midwives. Some individuals are appointed to play a leadership role for the unit–in the capacity as a charge midwife. Thus, a charge midwife is the leader of the maternity unit and is responsible for the operation of the unit over a specific period. Hence, their role in managing different aspects of maternity cannot be understated. Meanwhile, replete literature and empirical studies have focused primarily on midwives’ experiences in the implementation of RMC [11,16], without exploring the awareness level and exact role of other key maternity care providers such as charge midwives who are usually tasked with the mandate of overseeing the daily operations within the wards. This literature gap created by the dearth of empirical studies concerning the awareness and role of charge midwives in promoting RMC is the problem that this study seeks to address. We, therefore, explore the awareness and role of charge midwives in promoting RMC at a tertiary health facility in Ghana.

## Methods

### Design

An exploratory descriptive design was used. This study design was chosen as the aim of the study is to unearth and develop a deeper understanding of charge midwives’ awareness and roles in promoting RMC. Also, this study design allows us to be able to answer questions about why and how ward-in-charges play a role in RMC. Our study forms part of a larger study that seeks to explore RMC practice in Ghana [11,17].

### Study setting

The study was conducted in the maternity ward at Komfo Anokye Teaching Hospital (KATH) in Kumasi, Ghana’s Ashanti region. This hospital is Ghana’s second biggest and the only tertiary hospital in the Ashanti region [17]. With a bed capacity of 1,200 and midwife staff strength of about 381, KATH serves as the principal maternal referral health facility for the middle and savannah zones of Ghana [17]. It has thirteen (13) clinical directorates (departments) one of which is the Obstetrics and Gynaecology (O&G) directorate, which has four labour wards [17]. Additionally, there are 17 maternity units with each unit having one charge midwife.

### Participant recruitment process and sampling

In this study, charge midwives from KATH constituted the study population. We used a purposive sampling technique to identify and recruit the study participants. Prospective participants were expected to meet the following inclusion criteria to be eligible for participation: (a) they should be working in the capacity as a charge midwife, (b) they should have had at least one-year professional practice, and (c) they should voluntarily decide to participate in the study. During our reconnaissance visit to the health facility, one of the charge midwives volunteered to be a recruitment link for our study. As we were ready to start processes for data collection, the recruitment link was activated by contacting the charge midwives. There was no coercion as there was no power dynamics at play. The midwife who volunteered as a recruitment link only facilitated the process to identify other colleagues who were charge midwives. We provided the information sheet and informed consent form to be given to all prospective participants. We then granted the prospective participants two weeks to decide whether they would want to participate in our study. Signed informed consent was returned to us through the recruitment link. VMD and ABBM contacted those who had returned the informed consent form indicating their acceptance to participate in the study. During this contact, we discussed with the prospective participants their preferred day and time for the interviews and the mode of the interview. All of the prospective participants consented to have a face-to-face interview. The charge midwives who participated in the study had an in-depth understanding of RMC because they had participated in RMC training modules such as “alternative birthing positions, focused antenatal care, and empathetic and ethical communication with childbearing women” [17].

### Data collection and procedures

Two research assistants (RAs) were trained prior to the data collection. This training spanned two days. It covered the aim of the study and the procedures for data collection. We used semi-structured interview guides as the data collection tool. This tool was designed based on an RMC module (RMC-M) developed by Dzomeku et al. [11]. Although the charge midwives had already consented to participate in the study, we commenced our data collection by revisiting the objectives of the study and affirming the rights of the participants. We informed the participants about any possible discomfort, benefits, and compensation. Once we had received an affirmation from the participant, the RAs initiated an audio recording. We conducted the interviews at a date, time, and place of convenience to the participants. All the interviews were conducted at the premises of the maternity ward, KATH. A sample of the data collection tool has been attached as a (see S1 File). Our data collection began on 1^st^ May 2021 and ended on 9^th^ August 2021. On average, the interviews lasted for about 60 minutes. Data saturation was reached by the ninth interview, as there was no new analytical information emerging from the interviews. In all, nine charge midwives participated in this study. None of the participants withdrew at any point in the study. All the interviews were conducted in English.

### Ethical issues

We received institutional approval from the Komfo Anokye Teaching Hospital (KATH) Research and Development Unit (reference number: RD/CR17/289) and the Committee on Human Research, Publication, and Ethics (CHRPE) at the Kwame Nkrumah University of Science and Technology (KNUST) (reference number: CHRPE/AP/181/18). To protect the identities of our study participants, we anonymised all the transcripts and audio files by assigning pseudonyms to remove any personal information that can be traced to the participants. An information sheet containing the purpose of the study, procedures, possible risks, and benefits, compensations, who to contact, and affirmation of confidentiality, privacy, and autonomy, was provided to the study participants. We sought written informed consent from the participants. This indicated their decision to voluntarily participate in our study after having read and understood the terms of reference. We also locked the audio files and transcripts with an encrypted passcode to prevent unauthorised persons from having access to the data. The study follows the Standard for Reporting Qualitative Research (SRQR).

### Data management and analyses

After each day of data collection, the audio data was transcribed verbatim. The reason for transcribing on the same day of the interview was to enable the RAs to identify questions that may have been left unanswered during the interview. Subsequently, the RAs followed up on such transcripts and questions to get detailed accounts from the participants. After the transcription of the data, the transcripts were vetted and proofread by VMD and ABBM. When this vetting process was completed, the transcripts were made accessible to JO, who performed the initial independent thematic analysis. The transcripts were imported into QSR NVivo-12 for data management and analysis. JO began the data analysis by first reading all nine transcripts on three different days to familiarise himself with the data. Using the ‘nodes’ function in QSR NVivo-12, codes were assigned to the text data. Recurring codes that were similar were categorised to generate themes and sub-themes. Extracts and quotes from the themes and sub-themes generated were used to support the results of the study. We conducted member checking with three of the charge midwives who confirmed that the findings reflected their perspectives. The dataset analysed in this study forms part of a major study conducted in Ghana [11,17].

### Rigour and trustworthiness

We recognise the importance of rigour and trustworthiness in qualitative research. Hence, we worked to ensure confirmability and transferability. The study findings were transferable and confirmable due to the detailed description of the study methodologies and techniques. We did member checking with three of the participants to establish credibility; this was done one week following transcription and initial analysis of the data for the participants to confirm that the transcripts correctly reflected the interview material. No one submitted revisions or raised issues about the content and quality of the interviews in articulating their perspectives. After each interview, field notes were taken and referred to during the analysis, which included the participants’ nonverbal indications, worries, and interviewers’ reflections. The RA who conducted the interviews is a trained healthcare researcher with substantial experience in conducting IDIs and FGDs. The RA, on the other hand, does not work in the study settings and had no direct relationship with the participants.

### Patient and public involvement

Members of the public and the study participants were not involved in the design of the study. However, charge midwives participated in the conduct of the study as participants. The results from our study are intended to be shared with members of the public, healthcare professionals, and relevant stakeholders via scientific publications, lay reports, social media, and conferences.

## Results

Table 1 presents the distribution of the background characteristics of the charge midwives. Nine semi-structured qualitative interviews were conducted. The ages of the participants ranged between 33 and 54 years. Except for one participant, all the charge midwives had acquired a bachelor’s degree as the highest educational qualification. All the participants were married, except for one who was divorced. The lowest parity was 0 while the highest parity was 3. Concerning years of experience in practice, it ranged between 4 and 26 years.

**Table 1:** Background characteristics of participants.

Participant ID	Age	Education	Marital status	Parity	Years of practice
P1 (Ama)	40	Bachelor	Divorced	2	10
P2 (Abena)	36	Bachelor	Married	1	11
P3 (Adwoa)	33	Bachelor	Married	3	8
P4 (Akosua)	34	Diploma	Married	2	11
P5 (Akua)	37	Bachelor	Married	0	13
P6 (Yaa)	34	Bachelor	Married	2	13
P7 (Afia)	37	Bachelor	Married	3	12
P8 (Enyo)	54	Bachelor	Married	1	26
P9 (Kafui)	33	Bachelor	Married	2	4

Source: Fieldwork, 2021.

### Main findings

Seven primary codes were assigned and later categorised under two main themes, namely: charge midwives’ understanding about RMC, and the perceived roles of charge midwives in promoting RMC (Table 2).

**Table 2:** Emerging themes.

Themes	Sub-themes
Charge midwives’ understanding about RMC	RMC as showing dignity, respect and privacy
	RMC as women-centred care
Perceived roles of charge midwives in promoting RMC	Mentoring midwives and leading by example
	Show empathy and establish friendly relationship with clients
	Receiving and addressing clients’ complaints Establishing trust
	Monitoring and supervision

Source: Fieldwork, 2021.

### Charge midwives’ understanding about RMC

We began by exploring charge midwives’ understanding of RMC. The study revealed that charge midwives understood RMC from two main perspectives. The first perspective was that RMC is all about showing dignity, respect, and upholding the privacy of clients. The second perspective was that RMC was equivalent to women-centred care.

#### RMC as showing dignity, respect, and privacy

From the participants’ perspectives, RMC is primarily about portraying dignity and respect for and protecting the privacy of clients. By dignity, charge midwives opined that RMC is about making the client welcomed by speaking to them in a calm, tactful, and caring manner. According to the participants, RMC is not an event but a process and relationship that is characterised by providing equal opportunity for all clients who visit the facility for childbirth. This relationship is expected to be devoid of discrimination.

Abena asserted that:

*“My personal understanding of the concept is that concerning the clients we serve*, *when they do come to our outfit for care*, *we have to treat all of them as being equal*, *we cannot discriminate on the grounds that I know this person is this or that (status)*. *Whatever care a client’s needs*, *we must provide*. *We must not consider the person’s ways (behaviour or character)*, *we must not consider the educational qualification or anything of the sort*. *Whatever the client deserves that we give to her*, *let us give it to her so she can achieve the goals for which the client came to the facility”*. (Abena_P2_36 years).

Adwoa shared this experience:

*“When there’s care and dignity*, *confidentiality and privacy given to clients when they come to our facility*. *For me*, *I think that the dignity of the client should be respected at all times irrespective of how the client is behaving in the labour room*. *I believe that is exactly what respectful maternity care is all about”* (Adwoa_P3_36 years).

Ama opined that RMC means respecting the privacy and boundaries of the client:

*“If you approach a client to give her care and she feels she is not ready*, *if she tells you ‘I am not ready’*, *here*, *if you are not ready*, *we wait*, *as long as it is not an issue where delaying can prove detrimental to you*, *then we will wait (if delaying will be detrimental to you*, *then we won’t wait) but if we wait on the procedure and it cannot be detrimental to you*, *then we will wait*. *When you are ready*, *then you come and we do the procedure*. *Also*, *there is no shouting (at clients)*, *no beating (of clients)*, *she calling you for help and you refusing to attend to the call does not exist here”* (Ama_P1_40 years).

### RMC as women-centred care

The study revealed that charge midwives understood RMC as a women-centred care. To them, RMC is all about doing what the client desires. This women-centred care was argued by the participants to imply the inclusion of women who come to the facility for childbirth in the entire decision-making process. Thus, nothing about the woman in labour should be done without her informed consent. Opportunities are expected to be provided to clients to play an active role in the decision-making.

Yaa stated:

*“My understanding is that; respectful maternity care is all about the woman*. *You need to do everything that the woman wants*, *not what you want*. *It is more like the woman is the centre of the entire activity*. *It is about involving the woman in the decision-making process and ensuring that they make informed decisions not imposed ones*.*”* (Akua_P5_37 years).

Enyo also narrated that:

*“Well*, *in all you do*, *you have to make your mind that you will not step on the feet of clients and whatever you want to do for the client*, *the client will receive some benefits that are helpful to the clients themselves whether it is the cure of a disease*, *or if the client to deliver a baby*, *the client wouldn’t experience any negative feelings or thoughts concerning the duties you performed for her*. *That is how I understand the concept*. *So*, *essentially*, *it is all about putting your client ahead of any other thing*.*”* (Enyo_P8_54 years).

### Perceived roles of charge midwives in promoting RMC

We further explored how charge midwives understood their roles in promoting RMC services. The study showed that the role of the charge midwives in RMC included training midwives on RMC and leading by example; showing empathy and establishing friendly relationships with clients; receiving and addressing clients’ concerns; and monitoring and supervising midwives.

### Mentoring midwives and leading by example

One of the most recurring issues that emerged from our findings was the leadership role that charge midwives play in the promotion of RMC. The participants asserted that the charge midwives’ role is to lead by example. That is, the charge midwives initiated some activities that they deemed to be a reflection of RMC. This was done with the intention of leading by example. Thus, by exemplifying RMC principles of dignity, respect, and priority for privacy, charge midwives believed that their subordinates would imitate and follow that line of action.

Akua narrated:

*“OK*. *In that regard*, *what I do is that I make sure I lead by example*. *Therefore*, *those patients that…*. *though I am the in-charge*, *I don’t always sit at the table*: *I do work*. *When you get in here*, *I work and mingle with them like…So*, *what I do*, *I know juniors (staff)*, *they look up to seniors to copy*. *So*, *what I do*, *they look up to me to copy from me*. *Thus*, *I make sure that I am treating the patient with respect so that the junior staff who just started working*, *if she observes what even the in-charge is doing*, *she too can copy and do likewise*. *And from time to time too*, *I remind them of being conscious of how they deal with these mothers”* (Akua_P5_37 years).

Another participant also shared that they used their leadership role to assign new staff to older staff. This assignment aims to train the new staff on RMC principles to enable this person to adapt to RMC tenets.

*“I do not know about my predecessors*, *but if you are new here*, *I put you under the tutelage of someone I know to be very competent and the procedures and other things that go on here*, *you would be taken through them as an orientation*. *She will take you through them…*. *this is what goes on*, *this is how you should comport yourself*. *Well*, *I intentionally partner you with someone so you can be taken through all the protocols*. *If you have any problem*, *I will tell you ‘Report back to me’ so we can know what to do about it*.*”* (Enyo_P8_54 years).

### Show empathy and establish friendly relationships with clients

The participants from this study also asserted that the role of charge midwives in promoting RMC is to show empathy and establish friendly relationships with clients. According to the participants, they do this by informing the clients about all that she is likely to experience at every point of the labour process. Also, they performed sacral massages as a way of showing empathy to clients.

Kafui expressed her views that:

*“I try to calmly talk to such client and give her sacral massage if I’m the one taking care of her or if I assigned someone*, *I make sure the person does same*. *We tell her the implications of what she’s doing if she understands fine*, *if she doesn’t*, *we give her time and later attends to her*.*”* (Kafui_P9_33 years).

Similarly, Yaa contended that:

*“I care for the patient like a royal ‘someone’ because the person actually trusts me*: *that’s why she decides to come to me for the help or the care*. *So on the ward*, *I tell my colleagues that we should satisfy their interests first and care for them as if a patient is in labour*, *at times they are psychologically disturbed*, *you should have that at the back of your mind*, *you don’t shout at the patient when they start misbehaving or playing something in the wrong direction*, *you have to put them right and you must have…be ready to pull the long rope with them in the sense that the situation in which they are in is for the meantime*: *immediately they deliver*, *everything goes back to normal*. *So*, *we satisfy their interests first and allow them to feel comfortable*, *to feel at home*, *and then cooperate with our treatment*.*”* (Yaa_P6_44 years).

### Receiving and addressing clients’ complaints

As social beings, it is expected that there will be some deviance from the tenets of RMC. The participants reported that their role was to receive clients’ complaints about activities that can be categorised as disrespectful maternity care (i.e., shouting, beating, hitting, or slapping clients in labour). Their role is to address these complaints. From the accounts of the participants, such acts of disrespect are escalated to a higher authority to decide on what course of action to take. This role by the charge midwives serves as a way of deterring healthcare workers from acting disrespectfully to clients.

Akua expressed:

“the truth is that most of the time, the patients themselves do not normally come to complain, but it is the relatives who will come and maybe complain about one or two staff. So, when there are any complaints, I take them and then try to address it or escalate it to the higher authority at the facility so that we can deter others from disrespecting clients who come here to deliver.” (Akua_P5_37 years).

Afia also stated:

*“Some clients complain that some midwives talk harshly to them so as an in-charge I make sure to talk to the staff every morning before they begin their work in order to put them on their toes*. *Sometimes when the patient complains I call the midwife in question to ask about her side of the story so that we know how to handle the situation*. *The information I gather determines how I handles the whole situation*.*”* (Afia_P7_37 years).

### Monitoring and supervision

Our study revealed that monitoring and supervision were other important roles performed by charge midwives. According to the participants, this role was important as it allowed them to easily identify gaps in promoting RMC to clients.

Afia narrated that:

*“First*, *I have to supervise the work*. *Being a rotational nurse*, *student midwives or mine own staff (junior midwives and my subordinates) I will make sure they do the right thing as expected as respectful maternal care*.*”* (Afia_P7_37 years).

Ama narrated:

*“As an in-charge*, *I monitor the midwives under my supervision so that they treat women who come here to deliver with the utmost dignity and respect*.*”* (Ama_P1_40 years).

### Establishing trust

The study also revealed that charge midwives play a critical role in promoting RMC by establishing trust with patients. From the findings, it is indicative that charge midwives established trust as a precedence for women to open up about their feelings and perceptions about the care they are receiving. This helped the charge midwives to understand the concerns of the patient and provide appropriate remediating strategies.

Yaa shared similar views:

*“As an in-charge every morning you have to move from one cubicle to the other to ask of patient’s wellbeing and with that they feel free to tell you the complains about how the midwives treat them*. *Even among themselves they do communicate about the midwives so I think that establishing rapport with the women helps them to voice whatever they feel or how they are been treated*.*”* (Yaa_P6_44 years).

*“In my view*, *being an in-charge means that that I must establish a rapport with my clients in order for them effectively communicate their feelings about the care that I and my colleagues provide*.*”* (Afia_P7_37 years).

## Discussion

In this study, we sought to explore charge midwives’ awareness of and their role in promoting RMC at a tertiary facility in Ghana. We found two major themes that emanated from the data: charge midwives’ understanding of RMC and their perceived roles in promoting RMC.

The current study revealed that showing dignity, respect, and privacy and women-centred care were the two key sub-themes constituting charge midwives’ understanding of RMC. Health workers are expected to demonstrate the utmost professionalism in areas such as handling clients with respect, dignity, and protecting their privacy. Our finding is consistent with those of other studies [17–21], which stipulated that women seeking maternal healthcare services are to be treated with respectful care regardless of their background characteristics such as ethnic group, socio-economic status, and level of education among other. This also accords with our earlier observations, which showed that maintaining privacy and confidentiality; preserving women’s dignity, and not causing harm or mistreatment as the major component of RMC [20–23].

Additionally, charge midwives perceived RMC as a way of providing women-centred care. Our finding corroborates those of previous studies, which examined RMC [18,21]. As found in those studies and congruent to our findings, health professionals are of the view that RMC should focus on creating a safe and secure environment for their labour in aspects such as the provision of information and seeking informed consent, respecting women’s choices that strengthen their capabilities to give birth, and showcasing effective and sound communication [17,18]. This implies that the perceived understanding of RMC by the ward-in-charges could influence the quality of care rendered to the women, thereby encouraging health facility delivery.

Charge midwives in our study emphasised the need to train midwives on RMC and to set precedence in service provision to clients in aspects including but not limited to their understanding highlighted in this study as one of their key roles in promoting RMC. Studies across several jurisdictions have indicated the profound importance of in-service training in upgrading the skills and competencies of midwives and health workers in general [11,17,24]. This training can also take the form of workshops and seminars with practical demonstrations to give the requisite skills, competencies, and support to motivate the midwives to exhibit RMC and to change their perception and attitude towards handling clients [24].

Further, charge midwives are to show professionalism through showing empathy and establishing a friendly relationship with clients, which can be attained through effective and efficient communication, a key factor noted in several studies [18,21,25]. Women loved the verbal encouragement and support they got from midwives, as well as the emotional assistance they received while in labour. Charge midwives appreciated showing empathy to women and believed that talking and listening to the women was a crucial part of providing RMC. This also enables the charge midwives to receive and address clients’ complaints. Charge midwives role indicated in this study is consistent with existing literature [19,21,25]. Addressing the complaints should be devoid of stigmatisation and abusive words and gestures [26]. Naturally, women want to voice their concerns openly without worrying about being reprimanded. Even when the needs and expectations of women cannot be satisfied due to systemic issues that the provider is unable to address, straightforward communication may assist to mitigate the issue [25].

Moreover, monitoring and supervising subordinate midwives was noted as an important aspect of charge midwives’ role in promoting RMC. This also accords with our earlier observations, which showed that monitoring and supervising midwives helps to alleviate wrongful practices that hinder the attainment of RMC [27,28].

### Strengths and limitations

The major strength of this study is the use of detailed information on charge midwives’ understanding and roles of RMC. The involvement of midwives with expertise helps to provide rich data, which increases the validity and reliability of the findings. However, our study has pitfalls that need to be acknowledged. First, the adoption of the qualitative design limits the generalisability of the findings to all charge midwives. Also, the inclusion of only midwives in hospital settings could have introduced social desirability bias in the responses the charge midwives provided. Future studies should explore women’s perceptions about midwifery care. In addition, the authors did not pay attention to the barriers in performing RMC to clients. This is a limitation of the scope of the study and should be considered during the interpretation of the findings in our study.

## Conclusion

Charge midwives have a significant role in promoting RMC, which extends beyond only providing maternity care. The study concludes that policymakers and healthcare managers should ensure that charge midwives receive adequate and regular training on RMC. This training should be comprehensive, covering aspects such as effective communication, privacy and confidentiality, informed consent, and women-centred care. The study also underscores a need for policymakers and health facility managers to prioritise the provision of resources and support for the implementation of RMC policies and guidelines in all healthcare facilities. This will ensure that healthcare providers have the necessary tools and resources to provide RMC to clients.

## Supporting information

S1 File (DOCX)

S2 File (DOCX)

## Abbreviations

CHRPE: Committee on Human Research Publication, and Ethics
FDGs: Focused Group Discussions
IDIs: In-depth Interviews
KATH: Komfo Anokye Teaching hospital
KNUST: Kwame Nkrumah University of Science and Technology
LMICs: Low and Middle Income Countries
MDGs: Millennium Development Goals
MMR: Maternal Mortality Ratio
O&G: Obstetrics and Gynaecology
RAs: Research Assistants
RMC-M: Respectful Maternity Care-Module
RMC: Respectful Maternity Care
SDG: Sustainable Development Goals
WHO: World Health Organization
