# Supplementary material for: Charge midwives’ awareness of and their role in promoting respectful maternity care at a tertiary health facility in Ghana: A qualitative study
Source: PLoS One. 2023 May 15;18(5):e0284326. doi: 10.1371/journal.pone.0284326 (PMC10184897; doi:10.1371/journal.pone.0284326)
Supplement: S1 File — (DOCX) [file pone.0284326.s001.docx]

**Interview protocol for Ward-In-Charges**

**Demographic details:**

1. Age:
2. Education qualification:
3. Years of experience:
4. Parity status:
5. Married: A: Never married

B: Currently married

C: Previously Married

Understanding the Role of the Ward-in-Charges with reference to Respectful Maternity Care (RMC)

|  |  |
| --- | --- |
| 1. **Awareness of Respectful Maternity Care (RMC)** | Probe [ask them] |
| 1. Your understanding of respectful maternity care | Mention keywords or provide examples that show your understanding of RMC/ roles you play to promote RMC |
| 1. What strategies do you adopt to help women-in-labour whose actions (direct or indirect) may put baby and themselves at risk/danger? | Have you apologized to any woman after helping her deliver safely with any of these strategies? |
| 1. Have YOU seen or heard or have received a report that a healthcare provider (nurses, midwives, etc) treat a woman roughly, like push, beat, slap, pinch, physically restrain or gag them when delivering at the facility? | If Yes, provide some context: what triggered the action and what was your response?  If Yes, what actions did you take?  If actions were taken, what was the outcome (or what happened next)? |
| 1. Have YOU seen or heard or have received a report that a healthcare provider (nurses, midwives, etc) treat a woman without seeking her consent or attend to them without permission when delivering at the facility? | If Yes, provide some context: what triggered the action and what was your response?  If Yes, what actions did you take?  If actions were taken, what was the outcome (or what happened next)? |
| 1. **Your role as a ward-in-charge in promoting RMC** |  |
| **Privacy** |  |
| 1. How many privacy screens do you have? Are they adequate? | Do you have facilities or resources to provide privacy adequately? Tell me about it. |
| 1. How do you ensure that your ward has adequate privacy screens to protect the privacy of childbearing women? | Which units in the hospitals do you interact with and what form does the interaction take? |
| 1. What challenges do you face in ensuring that your ward has adequate resources and facilities to provide privacy? | What are your frustrations, and what are your responses to these challenges? |
| **In-service training for new midwives and existing staff** |  |
| 1. Takes your staff through the rights of the childbearing women | If you do take them through trainings on clients’ rights, what are your impressions on your staff’s response to these training/meetings? Do they perceive it to be routine and not take them seriously? Are all staffs under you obliged to be at the meetings? |
| 1. Takes staff through how to ensure that childbearing women are respected, and treated with dignity |  |
| 1. What challenges do you encounter in training your staff/new midwives |  |
| **Working with the social welfare department of the hospital** |  |
| 1. What do you do when it is reported to you that some new mothers cannot pay for services or any other concerns? | Who/which unit in the hospital ensures detainment in the facility if a client is unable to pay for the service? |
| 1. How do you resolve the discharging of clients who cannot pay for services? |  |
| 1. Do you work with the social welfare department in processing the discharging of these clients, and what difficulties did you have in working with them? |  |
| **Measures to receive reports of abuse from childbearing women** |  |
| 1. What reporting systems are available to encourage childbearing women to report abuse? | Have you ever received reports of abuse from childbearing women, and how often? What have been your response to these reports?  What are the types/nature of abuses reported, if any? |
| 1. If the systems are available, how friendly are they to all class of women (uneducated, adolescent girls, people with disability, people with chronic diseases (comorbidities), poor women). |  |
| 1. If there are systems in place to routinely monitor or investigate occurrences of mistreatment even in the absence of received reports from childbearing women. |  |
