# Supplementary material for: Charge midwives’ awareness of and their role in promoting respectful maternity care at a tertiary health facility in Ghana: A qualitative study
Source: PLoS One. 2023 May 15;18(5):e0284326. doi: 10.1371/journal.pone.0284326 (PMC10184897; doi:10.1371/journal.pone.0284326)
Supplement: S2 File — (DOCX) [file pone.0284326.s002.docx]

**Transcripts**

AFIA IC 29 MINUTES 16 SECONDS.

I: I’m very grateful for your time this afternoon in-charge. As I’ve explained do you

understand or you’ve a question to ask about our research?

R: I understand.

I: Meaning we can continue with the interview?

R: Yes please.

I: Agreed?

R: Yes please.

I: Thank you. In-charge please how old are you?

R: Forty (40) years.

I: Erm please what’s your educational qualification?

R: BSc Nursing.

I: Mm…please how many years have you practiced midwifery?

R: Ten (10) years.

I: Please ten (10) years? Okay.

R: Seventeen (17) years.

I: Please what year did you started working at Komfo Anokye?

R: Two thousand (2000) erm… February 2004.

I: Aha! Then it is seventeen (17) years. Okay.

R: Mm…

I: Please how many children do you have?

R: …(laughs) please I have two (2) children.

I: Are you married please?

R: Please no, I’m a divorcee.

I: Okay. Please how do you understand respectful maternal care?

R: To my understanding when a woman is in to deliver, she needs to be respected,

pampered and also have a sound mind as we’re here for her and not to treat her bad.

I: Okay thank you very much. Please as an in-charge at this Special labor ward what

measures have you put in place to implement respectful maternal care?

R: My number one role is to teach my nurses how to communicate with the patients

because of language differences. Also, they should take time to explain issues for them

to understand.

I: Okay thank you. What are some of the strategies you adopt to help women in labor

whose actions may put their lives and baby’s own at risk?

R: Please can you repeat the question?

I: Please I said what are the strategies you adopt to help women in labor whose actions

may put their lives and baby’s own at risk? For instance; when a patient in her second

stage of labor refuses to comply to instructions given to her what do you do?

R: We talk to the patient to comply to instructions else a machine would be used to push the

baby out. With this, some patient will ask for help and that is the assisted birthing.

Others there’s no option than to send them to the theatre because of less strength.

I: Hmm, some midwives said holding the nose of the patient to cease her breathe sends a

signal to the mother about how the baby is suffering. Therefore, they will gather energy

to push the baby down. Do you also do that at times?

R: To me is a bit cruel I don’t teach that. The patient is already in pain why then hold her

nose? At times is hungriness or thirst you can decide to give her water and encourage

her.

I: Okay. Please as you became an in-charge have you received a report about your nurses

or midwives treating patients badly as in slapping, beating, pushing, pinching or

physically mistraining or gagging them during delivery.

R: Yes, I’ve heard about it but not here. That was the olden days strategy of midwives

especially the unrolled midwives to get a patient to push down her baby.

I: Since you became an in-charge you’ve not receive any report that (in-charge quickly

answered).

R: Oh no I’ve not received any such report.

I: Okay. Has there been any report about your health care providers treating a patient

without her permission?

R: I’ve had some few reports but it has been addressed. The midwife might think because

she’s on duty she has access to you but I’ve informed them that they should always

prompt a patient attention before the next move.

I: Please how did the patient reported to you?

R: Oh, she appreciated our hard work when she was discharged and complained about a

nurse who helped her in delivery without her concern. I asked her how she was been

treated and she said it is her first time at the labor room and she was instructed to sleep

on a bed. All she heard was madam if you’ve to push, push though she was pushing

already but she later felt a hand has been inserted into her. She was confused and

screamed. I asked her twice if she was told that would be done to check the baby’s

position she replied no. I apologized to her.

I: What did you do to the midwife in question?

R: I called her and she explained the baby was in a wrong position but she apologized about

the behavior and I advised her to seek a patient concern next time. Also, during one of

our ward meetings I talked about it and reemphasize the need for that.

I: Please how many privacy screens do you have?

R: In our ward, every bed is curtained and even at the labor ward same so there’s much

privacy.

I: Meaning your screens are adequate?

R: Oh, more than enough.

I: Has there been a time there were no screens?

R: It has never happened here.

I: Oh really?

R: Special ward it has never happened.

I: Recently when I came I saw your screen has been changed and before that you were

already using one. What was the challenges when you went for the procurement?

R: Challenges was with management releasing money to buy for us but we argued that this

is a Special ward so the ward needs to be neat, clean and should be welcoming

for our patients. It delayed but eventually we were able to get it.

I: So how long did it take to receive it?

R: Finally, it took two (2) weeks to a month before releasing the money to us.

I: How long did it take to when you wrote to them?

R: To write to them and release money to us takes about a month.

I: Okay. In-charge please do you take your child through the right of the child bearing

woman? For example; do you organize in-service training to take your staff through the

right of the child bearing woman? Do you teach the women about their own rights?

R: Yes please. We do both in-service and in-house training for the staff.

I: Please how long does it takes?

R: Most of the time it is done when there are new staff which is used to be every two (2)

months. There were some changes so the new ones were trained.

I: Okay. Please with the new trainees normally what’s their response, do you see any

improvements?

R: They are always excited about it and so much improvements because I tell them here is a

special ward and not like the ordinary ward so everyone must be treated equal else a

patient can write a document about you. Though it might be the first time of some

trainees working at the special ward but is all good.

I: Okay during the training do you have someone who takes the staff through how to

ensure that patients are treated with respect and dignity?

R: Yes, that is what some of your research program did for some of the staff, and we have

one or two people coming from our side. We also do workshop for nurses, midwife in

general, the unit and the whole department to improve maternal care.

I: In-charge with in-service training what are some of the challenges you encounter in

training your new staff?

R: Some staff come on time others does not turn up at all because they complain of no

motivation except using it to renew their PIN. If happens like this then I have to go the

extra mile to explain to them that it is to add up knowledge. Basically, is about timing,

staff themselves and the resources for the workshop.

I: Okay, so please what resources do you think (in-charge quickly replied)

R: Resource materials. At times we need to provide soft documents, hard copies for them to

go through also flyers and posters are needed at the ward to remind them but all these are

hardly to come by.

I: Erm, in-charge, what do you do when it is reported to you that some new mothers can’t

pay for their services?

R: Here is a Special ward and we really talk to our patient because you coming here is a

choice you decide. It’s either you plan for the cost or you opt for your insurance which is

not the National Health Insurance. A man once complained he’s not aware of the amount

been charged her wife for delivery but was later resolved. Hardly does it happen here.

I: With this incidence, you explained to the husband or the couple resolved their own

issue?

R: The couple resolved the issue but we explained to him the wife was aware of the amount

to be paid but he said the wife did not inform him. He later made the payment.

I: You said before a patient is admitted here you communicate with her but has there been

an instance where you’ve to work with the Social Welfare department in processing the

discharging of clients?

R: No please.

I: What reporting systems are in place to encourage patients to report abuse? Let say a

midwife misbehaved towards a patient what systems are in place to encourage the

patient to voice their concerns?

R: We communicate with our patients when a doctor directs them here. First of all, if

you’ve a problem you can write it and attached the in-charge name and submit. Also,

after general handing over, I or my shift in-charge goes around and check on patients we

get reports from there but others will ask for your contact privately so they call and tell

you their problem. Lastly, is the Quality Assurance Office at the hospital where you can

tell them your grievances.

I: So, you educate every patient? Because as I came and delivered here no one gave me

such education.

R: Please I wasn’t here at that time and maybe the Quality Assurance Office was not set up.

As an in-charge as you’re going higher and learning you add more to what is done

already just to improve the system.

I: Okay. Please with all these systems in place have you received any abuse report?

R: Not abuse really but as I gave an example about a woman who complained about a

midwife who attend to her without her permission. It was through my regular checks on

patients that I got to know about it. I always introduce myself as an in-charge to the

patients though some do complain about the food that was served but to the extent of

delivery abuse no one has reported to me or the Quality Assurance Office.

I: Alright. How friendly are these systems to all classes of women? For instance; if a

patient is literate, adolescent, with chronic disease or disability are the systems friendly

to all?

R: It’s very friendly and accommodating. We smile to each other, I don’t discriminate

because I enter all rooms to check on them and handing over are done together every

morning by introducing ourselves.

I: Now in-charge what have you put in place to help investigate abuse even if a patient

does not complain about such. Let’s say the woman who reported the midwife kept mute

what would’ve been done?

R: You can not put cameras in the labor ward but we do encourage partners to be around

during delivery especially, the husband or mother. Patients talks a lot so they suggest

their preference and it is done for them during dressing. It’s quite a friendly unit and

since the hospital has not written any report to our unit concerning abuse I think

everything is going on well.

I: There are other labor wards aside this Special ward and you’ve been there what do you

think the hospital management should put in place to ensure respectful matters?

R: There must be enough resources and midwives on the unit in order to reduce workload

and prevent stress. Most patients at the labor room does not comply to instructions.

Imagine one (1), two (2), three (3) people who came for night throughout the night has

stood to deliver six (6), seven (7) and only one (1) patient waste time during delivery

you get tired because she’s not making work easy. One (1) patient is to a one (1) nurse

may not be achieved but at least if we can get a minimum of three (3) deliveries to a

nurse it will be good.

With the resources I’m talking about the screens and delivery beds for the unit and the

beds should be adjustable not ordinary beds. If a patient is lying on the floor you need to

be doing frequent monitoring of the baby. Listening to FH the nurse has to bend to do

the listening and this will not encourage the nurse to even do it. Not that the midwife is

not ready to do it but the situation she finds herself in. We’re in COVID era, PPEs

should be enough because you’ve to talk to the patient, embracing the patient you need

to get something to cover yourself well so that when during delivery and there’s a splash

you know at least there was some sought of protection.

If all these are available work would be easy. I don’t think any midwife is trained to

work badly but it is encouraging to enter the ward and see your patients are

comfortable. Definitely if you’ve enough monitors or some digital foetal heat monitors

we will do it. For me this is Special ward and at least we’ve two delivery beds and two

lying beds and we have more than two fetal heart monitors so we can afford to put

some one (1) person till the patient delivers but the other side they can’t afford to do

that. If all these things are put in place it will encourage the midwives to do their best.

I: Mm…thank you very much. Please is there anything I did not mention but you think

would be necessary for respectful maternal care especially in your unit.

R: You’re welcome but the only thing is that we should let our patient understand that

delivery is not done by only one person. I, the midwife will do my best so she also has a

part to play so far as the health of the child is concerned I will respect her, I will do

everything but if she goes against what she’s been told to do the end result will not be

good for us. So, it’s two (2) ways not only the midwife but as a patient you also have

responsibilities so far as your health and that of your child is concerned.

I: Lastly, as an in-charge what things do you need that would help you (in-charge quickly

answered).

R: Some few months ago, we had a lot of issues. Our delivery bed but as I talk to you now

we’ve two strong delivery beds. Electrical one that’s adjustable and can suite any

situation, our screens, our curtains all have been done so far so good. For some few

months we had a lot of issues but now the hospital has provided almost all of them.

I: Did it take long?

R: Oh! it took a while some of them especially the beds, it took about six (6)

months but they provided them.

I: So, when it comes to the timely provision of resources is there anything to talk about?

R: Oh! our unit is different.

I: Okay.

R: Yes, so I will say we get better responses than the other side.

I: Okay.

R: Yes.

I: In-charge, I’m really grateful for your time. Thank you

very much.

AUGE 1C

AUDIO LENGTH 37:05 (MINUTES)

I: OK. In-Charge, good afternoon. Pease, I am very grateful that you’ve agreed to have this conversation with me this afternoon. And just as I explained earlier to you about the studies we are conducting, we also want to understand the opinions and the experiences of the in-charges concerning respectful maternity care. Please, have you agreed to this interview?

R: Yes please.

I: OK. And please, In-Charge, how old are you?

R: Number of years?

I: Yes.

R: Thirty-six (36).

I: Professional qualification.

R: Tertiary.

I: Tertiary?

R: Mmmmm…..

I: OK. Please, when you started practicing as a midwife, how long has it been?

R: Eleven years (11).

I: Eleven years?

R: Mmmmm…..

I: Please, have you given birth?

R: Three (3).

I: Please, are you married?

R: Yes please.

I: In-Charge, please, when we say respectful maternal care, how do you understand the concept?

R: My personal understanding of the concept is that, concerning the clients we serve, when they do come to our outfit for care, we have to treat all of them as being equal, we cannot discriminate on the grounds that I know this person is this or that (status). Whatever care a client needs, we must provide. We must not consider the person’s ways (behavior or character), we must not consider the educational qualification or anything of the sort. Whatever the client deserves that we give to her, let us give it to her so she can achieve the goals of coming here.

I: So, let us consider a hypothetical situation where a very important person comes here while you are on duty to deliver, and at the same time, a pauper or someone not that endowed is also here to deliver. So, between these two clients, what is the care you will offer to them?

R: I do not have to discriminate. Both of them are the same kind of people. The only issue that may bring some distinction is when you consider…..but how do you even tell when someone is wealthy or not?

I: Mmmmmm…..

R: Aha…so I consider the issue as, I have come to work, what I have to do – normal routine – whatever extra duties I have to take on, that is what I need to consider. I will have to make sure that nothing entices that when I do evaluate this particular client, the perception is that she may have a lot of money and therefore I must treat her better. Or, that this one looks like she is poor. Thus, my understanding is that I am here to perform my duties. What I need to consider is that the client is given the care she needs. At the end of the day, it is the health of the baby and the mother that we are interested in achieving.

I: Well, being an In-Charge, what are the activities you do to promote respectful maternal care among the nurses under your supervision? Being an In-charge, how do you ensure respectful maternal care in your unit?

R: Oh….you said in reference to my staff, right?

I: Yes. Right now, you are the in charge, so as an In-Charge on the ward, how do you ensure that the patients who come for delivery are given respectful and dignified care?

R: On my part, my staff that I work with…

I: Uh huh….

R: …..I have to make sure that the needed respect that I have to give to them, I do give to them so that they wouldn’t take any anger or pain and transfer them on to the patients. So we start our homework from the home….this place that we all exist in, little by little, we treat our colleagues well. Ahaa…because if you put pressure on the staff you work with, and you do not treat them well, indirectly, whenever the staff is conducting procedures on the patient, they will not do their duties from the heart. So I make sure that, every day, that atmosphere exists (at the workplace). But, do not misconstrue this to mean when someone does something that should not be done, I will not reprimand the person. I will surely confront the person, but how I talk to the person to bring peace so that the working environment engenders productivity is what we have to do.

I: And then, what strategies do you adopt to help women in labor whose actions either directly or indirectly may put baby and themselves at risk? Imagine you have a patient and you are fully aware that this patient is full…..

R: ….that she is difficult….Mmmmm…..

I: …..please lie down, she says ‘no’, ….

R: ….she says ‘no way’….

I: ….please do this, her response is to not comply….so what strategies do you adopt to help such women?

R: Well, just as you’ve put, we have some people who are really troublesome. There are some people who are ignorant of the implications of the actions they are taking. There are those who, when you instruct them to lie down, will not do and will rather be throwing themselves literally on the floor, but if you take the time and patience to explain to her that maybe ‘Sister ‘Asomasi’ (Jane Doe), your behavior will not help. If care is not taken, your nine-month journey will come to naught. Your antics can cause an injury or even death to your baby’. So, if you exercise patience and explain things adequately to her, if she is someone who is cognizant of her own actions, she will eventually be stunned and that can lead to her stopping the bad behavior. When it comes to this stage, it has nothing to do with screaming at people because there are those who will never respond to your shouting. She can even refer to you as having no patience for the job, or something to that effect. But if you get the patience to explain to her, she can go like ‘really?’ Then she will stop. And you can have the time to support her along the way so she can deliver safely.

I: OK. Well, when it comes to the time when they are delivering, there are those who do not cooperate….

R: …and push.

I: Ahaaaa…..she may put her thighs together and such behaviors, she doesn’t want to push…..

R: Oh, you just let her know the implications of her actions, that if the baby stays too long in there, it may become asphyxiated or such. Sometimes too, you can exhibit an example to her. For example, if you pinch and hold your own nose closed, and explain that the baby’s head is turned downward, and the nose is closed as if something is blocking it, so the longer you wait, the longer you are preventing the baby from breathing, so if you try harder and push……sometimes, when you do explain things, it really works because there are those who never would push otherwise, but the moment you say such things, they get frightened into pushing as they do not want their nine-month journey to be for nothing. So she will try harder and push and the baby will come out.

But if you try and the client doesn’t budge, then you call for more hands, and if it has to take a vacuum, then they will have to conduct that for her.

I: Mmmmmm…..so, when you talked about holding the nose (to hold the breath), were you talking about your own nose or the patient’s?

R: I use my own as an example, that she should observe if I hold mine like this (indicating), it means the baby is being deprived of air. Sometimes too, you can illustrate to the patient by holding her nose, not in an angry way, but in that, she can observe if she stops her breathing for a second, that is exactly the same way the baby is behaving by being stuck in there. Therefore, when this is done, she will see that it is not comfortable, not that you are trying to beat (abuse) her, but you are demonstrating to her.

I: So In-Charge, let’s say you have held her nose to demonstrate to her, after the delivery, are you able to apologize to her that maybe it was because she was in the process of delievery…..and because she didn’t want to push, that was why you held her nose….

R: Well, it wouldn’t be done in anger or a quarrel? You didn’t do that in anger, but in all, you are trying to show her that this is what is going on because some clients are very difficult….

I: Yeah…..

R: ..so if you do not do something like that to make her understand that the baby is struggling, there are those who will never cooperate. Uh huh…I know it is not right though, but at least when you do that, it makes her realize the severity of what you are saying. I know previously in the labor ward, clients were slapped but all such things are in the past. For this, you’ve not abused the client but it shows her that for this short time, maybe you tell her ‘do you realize this is how your baby’s nose is? I want you to realize this is exactly how the baby’s condition feels, so you know you must push. When some people come to the second stage here, they do all sorts of things. When you advice, they will never pay you any attention, claiming they are in pain. Meanwhile, the baby is on its way and whatever you do to block the baby from coming out doesn’t help the baby.

I: OK. So, In-Charge, as an in-charge, have you seen or heard, or have you received a report that a healthcare provider – nurse or a midwife – treated a woman roughly like beat, pushed, slapped, pinched, physically restrained or gagged them when delivering at the facility?

R: I’ve not heard anything of the sort here.

I: You’ve not seen or heard anything like that here?

R: No.

I: What about where you were previously? Where were you previously?

R: I wasn’t in the labor ward.

I: You were not in the labor ward?

R: No.

I: Oh OK. OK. And you’ve not heard anything of the sort here?

R: By the grace of God, we do not do such things here.

I: OK. But, maybe, while being here, you may have heard that maybe such things do happen on the labor ward or something like this? You’ve not heard anything of the sort?

R: No, I have not.

I: OK. Have you seen or heard, or have you received a report that either a nurse or a midwife treated a woman without seeking her consent or attend to them without permission when delivering at the ward? So, please, being the in-charge, it is possible a patient approaches you….or a nurse or anybody can approach you that ‘this particular nurse, please advise her because when she is about to do a procedure, she puts on a frowning face…..she will not exercise patience’…..

R: For this, I have experienced that.

I: Ahaaa……so, can you explain that scenario? What was the context? What was the situation like?

R: Oh..the patient told me that….you are talking only about labor? For this was a CS…..

I: On the ward….

R: On the ward…OK. She said she complained of pain to the nurse, but the nurse was trying to indicate that the last time she gave her pain medication wasn’t that long ago, so, it was like ‘I’ve given you pain killer within the last five (5) minutes and you are still complaining of pain’. It is not a case of the nurse insulting the patient, but it was her asking the patient ‘Oh, but it hasn’t been that long since I gave you the medication and you are still complaining of pain’? But what is of a little concern here is that, the nurse should have known that each individual has a different threshold for pain, so it caused a little problem for the patient. Thus, it led her to report the following morning that when she reported her pain to a particular nurse, her reply was that she’s ‘given me medication already, so I cannot come and complain that early about pain, and as a result, she ignored me’. This incident didn’t go very well (per standards) so we called the nurse and had a talk with her, pointing out the fact that every individual feels pain differently, so next time, if there is a similar incident, she should access the pain carefully. If it is beyond her capabilities, she should just call in the team on duty and if they have to change the pain medication, then they should. So, this is what transpired.

I: OK. So when you did this, what was the outcome? The nurse?

R: Oh, the nurse went and apologized to the patient…..

I: OK.

R: Uh huh…..that she was sorry for acting that way.

I: OK. Ah, In-Charge, please, in your ward, when we talk about the privacy screens available to you, are they many? Are they enough for you?

R: But we do rather use partitions here?

I: What do you use?

R: Those….(indicating), these curtains.

I: OK.

R: Or? When it comes to that, we do not have any. The screens….we don’t use them here…..

I: Oh OK, so in here, you do not…..

R: You know, this place is partitioned already….one person doesn’t see the next.

I: OK, so here, it is not privacy screens but rather curtains.

R: Mmmmm….

I: OK.

R: It is even more comfortable than screens.

I: But, are they adequate? These, the screens that you have, are they adequate for you?

R: Screen?

I: Eh….the curtains!

R: Yeah, everywhere is curtained…..

I: So they are adequate for….

R: …with the exception of side wards’ entrance, and even with that, sometimes we use the door…..it is adequate. All the four-in-one rooms, there are curtains in there. It is the side wards’ entrance that the curtains….uh huh…

I: That place is open…

R: That is the open space, but if you open the washroom’s door to cover it, nobody can see the inside, and as time goes on, moving forward, it is my opinion that we must do something about it, like getting a paper to cover the back, it would be nice.

I: Oh, OK. And then, recently when I was on a visit here, I realized you’ve gotten new curtains. So how do you ensure that your ward has adequate privacy screens, that is the curtains, to protect women who come in during labor?

R: Well, the curtains are the very thing we use to ensure privacy, so we make sure that they are in place on a daily basis. If you leave it just there, whenever another person is passing by, the person may see the nakedness of the client. Therefore, there was even a time when the curtains here were too old, and thus, we requested for new ones. And that is the reason why you see the new ones wonderfully put in place to make the ward so beautiful that got you talking.

I: Mmmmmm….and then, as an in-charge, what challenges do you face in ensuring that your ward has adequate resources and facilities to provide privacy? What challenges? You were saying that you ordered for these curtains. During the process, what were some of the challenges you faced?

I: (14:55-:59) sometimes, getting the curtains on time is an issue, but once we put in our request, the curtains eventually arrived. It took a little while though.

I: How long did it take? …… it took a while, right?

R: Yeah. It took some time. Let’s say it was for about a month even, before we received the new ones. Yeah. Though, once in a while, they get dirtied, so we will pull them down and take them to the laundry to be washed. Even in those instances, it depends on the state of the ward; if the place is less busy, we take them down and as fast as possible…..even when that is the case, you have to get a little money to incentivize the person running the laundry errand so he/she can bring them back early enough.

I: OK.

R: So that we can get them to provide the privacy.

I: But In-Charge, your new midwives and the current midwives who are on the ward, do you take your staff, do you organize an in-service training for them during which you teach them the rights of the child-bearing woman? So it could even be somewhere else apart from your ward, maybe the hospital organizes these….how do you ensure that, you know, you allow those new and current midwives take part in that training?

R: Well, once in a while, we will get a memo that a workshop is being organized. In that case, we select some people from both the new and old ones, and they attend it. And when they come back, the new trends that are in vogue, we share and they talk about them for all of us to know, and the new changes that need to take place and for us to work with, we share them all. So we assign people to attend – we all can’t attend at the same time though – and when they come back, they give us feedback…..

I: Mmmmmm…..

R: …and we also use it to improve on our work.

I: So, for instance, last year and the previous two years back, the respectful maternal workshop that we undertook…

R: Mmmmmm?

I: ….after your staffs attended the workshop, what are your impressions of your staff response to these training?

R: It has been a wonderful help.

I: In what way?

R: Aww…right now, you see the difference that even when the patient comes in and, excuse my language, misbehaves, we consider it as an extension of the pain she may be experiencing and so – we even know that already but it (the workshops) have given us a deeper appreciation of it to the point that we now understand that no matter what the patient does, it is incumbent upon you the health worker to exercise patience. And the things you need to explain. Even if you do this and a particular client is not cooperating, you just give her a little chance. Even if you have to call in the doctor after a while and let him/her also say a little bit – we all bring in our little bit – I know that it is not always by force to advice someone that this and that might not be good for them….it is possible they will not listen to you. But in my opinion, perseverance is essential – the more you talk to the patient, the patient will calm down, no matter who she is. You just continue to give her encouraging messages that the baby she is bringing into the world is cause for celebration, and some of them will listen (you will get through to some of them). Well, I can even say all of them will cooperate with you (eventually)….

I: Mmmmmm….

R: …and whatever is beyond you, when the pain is too much for the client, you call her doctor and maybe the doctor can intervene with an epidural or something else and she will calm down.

I: Please, whenever there are some meetings, do you get opportunities? Maybe, do you allow all your staffs to attend such meetings?

R: We allow some of them to, as I said earlier. You can’t allow the whole ward to attend…some will go and others will stay behind. When they get back, they bring back feedback and we all share and improve on the work.

I: Well, and what about within the unit itself, do you offer training to your staffs to train them, in order to ensure that child-bearing women are respected and treated with dignity? Sometimes when you do your meetings, do you educate them?

I: Yeah, we routinely do this. We do talk to each other about these things. Although because of the shift system associated with nursing, we all cannot meet, but at the end of the day when we do meet, we try that the shift that is actually meeting, the things that we have to do to improve upon our work, we talk about them so that the work schedules can flow seamlessly so that this brings peace.

I: OK. Well, still on the training, what are some of the challenges…let’s say you may want some of the midwives or new nurses under your supervision, left to you alone, to go for a particular training, but it may not be possible. What are some of the challenges you encounter in ensuring that you train your new nurses and even the current staffs? What are some of the challenges?

R: Well, we can talk about the resources…the resource personnel you may need to educate the staff, there are times they are not easy to come by as to, for example, you’ve identified a challenge on the ward, for instance a CTG machine, there are a few staffs that may never have used one before, it may be the first time the staff is stepping into the ward. Fine, even though we may be teaching the new staff little by little the things we do on the ward…the normal ones are easier for them to pick up easily, but how to interpret it (the CTG), for that, we need an expert to train new staff on this so that at the end of the day, if you have printed out the CTG results for someone, at least, a midwife should pick it and be able to read it to understand how the labor is progressing and is the outcome good or bad? Or the fetal heart rate has gone up too high? The rate at which the heart is beating, is it normal? These, all these things are challenges that at least, if we get the personnel to support us and educate us, it will help so that the work, we are much more closer to the patient, so we can know what to do, to intervene as fast as possible so this can positively impact patient care.

I: Uh huh…and then, as an in-charge, what do you do when it is reported to you that some new mothers cannot pay for services or any other financial concerns? Let’s say someone is here to deliver, and when it is time to be discharged, you get informed that ‘In-Charge, the woman’s amount is not sufficient’ or she doesn’t have the requisite amount to pay for the services. In such an instance, what do you do?

R: Well, in here, we have not encountered this challenge. Ideally, anyone who comes here, their doctors first instruct them to come over and make enquiries: you find out how much the bill will come out to be before you come. But once in a while, you will be here and an emergency arrives from the Accident and Emergency department down there (indicating)…

I: Down there, OK.

R: Down there, and the client would be brought up and you would be told ‘labor’. When such people give birth, if we ask you after delivery and you cannot afford it, we allow you to go to the labor ward for discharge. We do not worry ourselves exceedingly to try and force you to pay. You must understand there are people who were not, ideally, coming to this place….

I: Mmmmm….

R: …but circumstances brought her there…so we can conduct the delivery, and when we are done, we allow the client to go either to the labor ward or sponse (spelling? Sponsors?? Spont for Spontaneous ?) And if you have insurance, you pay for it, because, right here, because it is cash we accept here, and the amount is substantial, so if something like this comes to our attention – if the client is not intentionally coming here and is mistakenly brought here by a taxi and maybe there is a head in vagina, we will conduct the delivery but we do not charge her with the bill of this place. We take it to the other side. Well, it already happened. Let’s say I am a midwife and I inadvertently meet something like that somewhere, it doesn’t mean by all means since the client ended up at the Special Ward, we should charge her as such. When we are done, the appropriate route…if she had made up her mind to go to the A Block, we take her there and she goes through the normal procedure and pay with her insurance. That is how we handle such issues. In here, so far since I’ve been here, I’ve not witnessed any situation in which anyone who cannot pay being forced to pay.

I: Oh OK. OK. So, you’ve not really gotten any experience with the social department since you’ve been here?

R: ….I’ve witnessed, that is what I am recounting, but I have not taken anyone to the social department with the issue that the client cannot pay for the bill. Here, anyone who comes here is ready to pay. So they come, and they pay. That is all.

I: In-Charge, what reporting systems are available to encourage child-bearing women to report abuse? Thus, the women who come here to deliver, what mechanisms have you put in place so that in case a midwife do not treat a client well, the client can come to you to report the matter, that maybe ‘In-Charge, the treatment that was meted out to me by this particular midwife did not meet my expectation’. What are the resources you’ve deployed in this regard?

R: When it comes to that, we’ve so far not done anything in that regard, or provided a number that if you have a grievance, you should call. This is because of the relationship between us and the clients when they are here. Even if something untoward happens, most often than not, the client will confide in one person and she will report that ‘Oh, since I’ve been here, you’ve treated me very well but this particular person did something to me, and I didn’t like it so you guys should talk to her and advise her so she can change’. But, once you talked about this, my mind has been brought to the fact that, at least in a month, if we create something like a notice that this midwife is the one on duty, so if you have any concerns, call her. If there are any private issues, you can talk it over with her. I believe if we put our sight in that direction, it can help too. Because there are some people that even if they do have issues, out of fear, it is not everything they are willing to talk about.

I: Mmmmm….well, have you ever had a report like that before?

R: I was talking about a patient complaining the other time about having pain and a staff did not treat here well? That was the only incidence that so far I have encountered.

I: And that one was about the way she was spoken to?

R: It was about the manner she was spoken to. She expected that after she told the nurse about the pain, even though you’ve given her medication, you must realize that she is still in pain, and that is why she is talking about it, and not because she is being intentionally difficult. Uh huh…so, you must understand this is about two (2) things: the nurse feels she has given out the pain medication to you, and the duration for the medication is this long, and it has not even been an hour, so why are you complaining?

I: Mmmmm…..

R: So this involved two things, but in everything, it was the nurse who should have understood that you cannot measure pain threshold. So she should have first listened to the patient before she spoke, so things didn’t go very well. We thus talked to her.

I: OK. You were talking about the fact that the way you treat the clients when they come here, if they have any problem, they feel comfortable coming to talk to you guys about it. Well, that mechanism, because the relationship you’ve created with the clients is good and that is why they are able to approach you and talk to you. So do you think this strategy that you utilize, is it friendly to all classes of women? That is, there are some clients who are uneducated, teenagers, people with disability and people with chronic diseases and other co-morbidities. Let’s say a client is here with HIV, and then poor women…..

R: For these, we try. Like, when it comes to the Special Ward, when the patient comes, we know how to receive her at the front desk. We make you feel at home….

I: Mmmmmmm….

R: You know, some people are tensed, it is their first time coming to deliver a baby, she’s heard a lot about labor, about it being frightening, so you the midwife she is meeting at the front desk, if you do not welcome her with a smile and open up to her, you’ve already allowed the fear to penetrate her being. So we make sure that whenever anybody comes here, you must be cheerful, open up to them and smile, welcome her, and if she came with her husband, welcome him too. Therefore, we do receive them in a very good way at the entrance so they will feel comfortable. And then also, through the orientation that we will give you when it comes to admission, we thoroughly explain to you what will go on. Some people are not lucky: their doctors may not have fully educated them on what labor is. So at least during admission, if she is stable and not in a stage where the observation tells us that she is not in serious pain, that is, if she is in a latent phase, you can talk to her at length for her to understand the processes. But some clients get here far advanced in labor, so that it is very difficult for her to even hear what you are talking about properly, you know. So that is one challenge but mostly, when we try, we are successful.

I: Well, aside these one, what else? You know there are some patients who cannot come to you and let you know that ‘In-Charge’, maybe this and that is my problem, or this particular staff did this to me, and I am not happy about it. So what are the procedures you’ve put in place, that even if the patient doesn’t come to report to you, you as an in-charge will recognize there is an occurrence of abuse or mistreatment ….

R: Oh, well, sometimes, our own colleagues…we do have individual differences….

I: Uh huh…

R: If I am around and a colleague, though, in front of the patient….well, it isn’t one staff that do take care of a patient? At least, two or three (2 or 3) will be there, and all of us cannot agree that what you did to this patient is great, so we will not say anything to you. Sometimes, one of the staff can come to me and say ‘Ma, these days, Madam Asomasi (Madam Jane Doe) what she did to that patient wasn’t right at all, Ma, you need to talk to her. It is bad’.

I: OK.

R: Because, here, we know that in the Special Ward, we treat patients exceptionally well. So, among ourselves, if a colleague does something unethical to a patient, another colleague can call you out that what you did was not good though we might not say it directly in front of you, but we can report it to the in-charge that this and that went on, so ‘Ma, talk to her’. Or, that whatever someone did wasn’t good at all, uh huh…we make in-charge aware that our colleague did not behave well towards a patient, and we are not happy about it. And in this ward, everyone knows the character of everyone else, and even the clients with us know so-so-and-so is like this, Nurse A behaves this way, and Nurse B is like this, and Nurse C is like this, uh huh….so amongst ourselves, we are able to reveal certain things and we come to the conclusion that this person needs to do this….and you know that in nursing itself, when you do something, sometimes, when you report a certain behavior about a colleague, it seems you hate the person, it gets to a time when it is what it is…the in-charge herself knows who people are, sometimes, she can go to our leader and demand that ‘this particular staff is giving me problems here so I cannot work with her’…..

I: Mmmmmm….

R: ….and sometimes because of fear, someone can get to the in-charge but the in-charge cannot say or do anything about the issue. It is possible she might be afraid of the staff in particular (who is misbehaving) and cannot categorically reprimand her for doing something bad and the offending culprit will continue to engage in the bad behavior, but due to maybe some fears, the in-charge might not be able to say anything about it. So, this is also a challenge. Well, you being a leader, sometimes you face all these people….some are recalcitrant – there are those who take no heed even when you advise them - ….

I: Uh huh….

R: …so sometimes, a particular in-charge with such a staff may take the matter up to a superior and demand that maybe ‘Superior, this member of my staff, the attitude she portrays on my department is not helping me, so take her out of my department’. Sometimes…but at the end of the day, if we do not talk to the person to change her attitude, won’t she continue to behave in the same manner?

I: Mmmmmm…..

R: So this is also one of the challenges that are difficult at the workplace. But no matter what you do, as a leader, the way you will resolve conflicts and the way you manage yourself, you can calm nerves down. So it is equally important for us the leaders, when there is an incident, the way you go about solving it, or the way you handles issues can also lead to the subordinates you work with changing their attitudes. So, when everything goes around, it comes back to the jurisdiction of the in-charges a little, because, in some instances, because of some favors and such, giving of unnecessary gifts that are given to us, when a person does something so bad, instead of reprimanding the person, you cannot because of a gift. You would be mute. So let us be mindful…we also need advice amongst ourselves. Yeah.

I: So In-Charge, all the things we have discussed about respectful maternal care, is there something I did not touch that you may want to add to the discussion? Or do you have any recommendation, something you may want to add to the conversation.

R: Mmmmm….OK, well, right now, I am aware the patient has her rights; it is what she prefers that you must do for her.

I: Uh huh….

R: Today, people are enlightened. Someone might come and because of the pain associated with labor and delivery, the client can opt for an epidural. ‘I do not intend to go through the pain’, and in this case, if it was left to my judgment, the doctors also must talk to the clients to let them be aware of the need for the epidural; both the positive and the negative sides (implications). There are those clients who will always opt for it no matter what you do, and it is not that the epidural is bad – epidural works for some people - ….

I: Mmmmmm…..

R: ….if the monitoring and everything proceeds smoothly, someone can take the epidural and still safely deliver. So, in my estimation, it seems we the nurses only consider the negative aspects of the epidural, the midwives are for epidural (34:52-:54). Even if a patient comes and demand for an epidural, the sort of things the staff will say about the epidural….

I: Well, what are some of the negative aspects of it?

R: Well, sometimes, because it slows down the labor process and such…so someone may say that ‘what is your purpose for taking the epidural? The outcome of the epidural is not a good outcome’ and you may discourage the patient. But when it comes to this, each individual and what they may prefer….if the client takes it, the doctor has already informed her that ‘if I give you the epidural and it fails, it may lead to CS and other things’. Ahaa…for instance, ‘due to the epidural, the fetus inside is distressed, or the baby is distressed but the time is not yet due for delivery’ and if we must intervene…well, you explained all of the scenarios to her and she accepted them.

I: Mmmmmm….

R: Uh huh….therefore, we can incorporate this too. If the patient comes and she informs us of her preferences, we must endeavor to give her what she wants. We must not discourage her, or anything of the sort. And sometimes the relatives of the patients too, they are sometimes troublesome.

I: Uh huh…..

R: And in all these, the ANC plays a part. If they (clients) are advised there….especially here in Komfo Anokye to be precise, in some cases, the client is a sibling of a staff, she is here to deliver, and she would be inserting herself (the staff) into everything, and if you even ask her to excuse you, she wouldn’t understand, and a whole lot. So, I see it as if we talk to our clients very well from the scratch and they understand certain things, they will appreciate the care we give them a lot more. Uh huh….

I: OK. Please, is there anything else you may want to talk about?

R: Right now, I have exhausted all the points, so by God’s grace, on a different day….

I: Oh OK. Then In-Charge…

R: I am going to think about things, so when you come back on a different day, whatever we may have to add on, we can.

I: I hear you. Thank you very much In-Charge, for having this conversation with me this afternoon.

R: I thank you also.

CONY IC 25 MINUTES 30 SECONDS.

I: In-charge good afternoon.

R: Good afternoon.

I: Thank you very much for your time. In-charge please how old are you?

R: I’m thirty-three (33).

I: Your educational qualification?

R: Tertiary.

I: Years of midwifery experience?

R: Eight (8).

I: Eight (8) years. Please how many children do you have?

R: I have three (3) children.

I: Wow.

R: Yes please.

I: In-charge please how do you understand respectful maternal care?

R: When there’s care and dignity, confidentiality and privacy given to patient when they

come to our facility.

I: Being a shift in-charge what are the things you do that promote respectful maternal care?

R: You welcome the patient into your facility you introduce yourself as the midwife on

duty then you ask her name and her reason for coming there in a friendly manner so that

the patient will feel comfortable and tell you everything. Make sure you explain every

procedure to the patient before you begin an examination so as to gain her cooperation

and also try to ensure privacy.

I: What strategies do you adopt to cater for patients that does not comply to instructions?

R: We try to talk to the patient ask her what she want it can be dehydration so we give her

water in order to help her save the baby. Sometimes, I cease their breath for a while to

show them how their baby is suffering so that she will cooperate and you the midwife

should try to monitor the fetal heart rate (FHR) and hydrate her to keep the baby safe.

I: Have you had a situation that you adopt these strategies and apologize to the mother after

delivery?

R: Oh yes, I’ve had such an encounter. A patient came and wasn’t complying to

instructions it was her first time in the labor room. She stood from the couch and with

the help of the doctors we got her back on the couch and the doctors hold her legs and

hands but still she was not pushing. We shouted on her and after the delivery I told her

she could have lost the baby even the baby came out weak but she realized her fault.

I: Have you heard or has someone report one of your midwives to you about how badly she

treated her?

R: Oh no there has not been any report about that it was only one patient that complained

that we have scolded her but luckily the team of duty doctor was around so he told her

the scold gave her the eagerness to push else she wouldn’t have push down the baby.

I: Has it been reported to you that your midwives don’t seek consent from patients when

performing an examination?

R: I have not witnessed any and here we explain procedures to patients before performing

any examination. To attend to a patient and just turn her around or begin examination no

we don’t do that.

I: You don’t do that and no one has ever complained?

R: No, I’ve not witnessed and no one has ever complained.

I: In-charge please how many screens do you have here?

R: Screens we have…we have one (1) best screen.

I: Is the one (1) adequate for the ward?

R: No but we have two (2) faulty screens.

I: How do you ensure you have adequate private screen in your ward?

R: The screen we will use will help us ensure privacy. We monitor two (2) patients in one

(1) cubicle so ideally, we have to get enough screens. Now that is not available if the

patient can walk she walk to where the screen is to ensure privacy if not in the

lithotomy position we cover her knees with cloth for privacy.

I: Where do you request for screens?

R: We write to the business manager to sign then to stores. If we follow that procedure and

is available they give to us if not available they will let us know.

I: When it comes to how you provide privacy as you’re saying you’ve only one (1)

functioning screen, not only screens but curtains too and also your labor ward is opened

what challenges do you face in providing privacy for patient?

R: We follow procedure to request for screens and the faulty ones we write to the

maintenance. If they can repair they do it for us and with the curtains we don’t use some

here we use only screens.

I: Okay.

R: Yes, we use curtains at the second stage so for the first and fourth we use screens. If it

gets faulty we write to the maintenance or to the stores for new one.

I: What are some of the frustrations you get when you request for new ones and is not yet

in?

R: It is very frustrating when a patient is here and there’s no privacy and doctors will be

moving up and down but there’s nothing we can do because we don’t have the

equipment to provide privacy.

I: In-charge do you have in-service training that you do for both new and old midwives to

educate them on the rights of a patients?

R: Yes, we do.

I: You do. Is it routine or you do it yourself?

R: For the old ones we organize it monthly but when there’s new staff we organize an in-

service training to make introduction and show them the environment. Not every month

but once, twice or thrice within a year.

I: It is supposed to be attend by all staffs?

R: Yes.

I: Oh okay. After training do you see any improvement in the staffs?

R: After training we see a lot of changes. It can be the person doesn’t know so after the

training it prompt our attention and it helps us all.

I: Please do you organize training to take staffs through the way to ensure that patients are

respected and also treated with dignity?

R: Yes.

I: What challenges do you encounter in terms of getting resources and stuffs to organize

the training?

R: One thing is you wouldn’t get all staffs to attend because some will be attending to

patients, others maybe on annual leave and others maybe off duty. These are the minor

challenges we face but we record the meeting and upload it on our page. We have a

page.

I: Okay. So, you are saying you’ve a page.

R: Yes, we record everything that goes on and upload it on our page so if you have any

submission you add up.

I: What do you do when some patients cannot afford their bills?

R: In the hospital policy, we have to call the social welfare so how the patient is going to

pay is up to them.

I: In this hospital, who ensures that a patient must be detained if she can’t afford her bills?

R: It is the responsibility of the in-charge, social welfare and also the staffs on duty.

I: Okay.

R: If for some reasons a patient can’t settle her bills we have to cater for her because she

can escape. So, we quickly inform social welfare so that they take her particulars and

how she’s going to pay if not we contact the patient’s next of kin. It is our responsibility

to detain her if the social welfare tell sort everything out we can then allow her to go.

I: What are some of the challenges you have in working with social welfare from your

experience?

R: Right now, we don’t have any problem with them just that it takes time when you call

them but won’t take a day or two (2).

I: Oh okay.

R: It takes maybe hours for them to come. Aside that when they come they do the needful

and if they ask us to discharge the patient we do.

I: What measures has been put in place to ensure that patient can report their issues

concerning how they were treated?

R: We create friendly environment for them to know we are here for their wellbeing and

that of the baby. If anything worries them they can inform us and we ensure them that

what ever been told to us unless they give us permission to involve a third party and

because of that they are free to call us if they have any complains.

I: Has any patient complain about a staff behavior to you?

R: Oh no. No one has complained about a staff behavior or something she has done.

I: With the friendly environment, may not be you but with other colleagues do you think

the environment is friendly to all patients irrespective of being educated or not, teenager

or adult?

R: Friendly environment in our profession is to make the patient feels free and open up if

she has any complains. Physically, emotionally and financially if you can help the

person that is fine. Some people come here to settle bills for others so if I have a

detained patient I can call you out for yours to be settled. That’s how we have created the

friendly environment.

I: Aside these measures, is there any measure that has been put in place to monitor staffs

on how they treat patient without the patient confronting you?

R: Here we don’t have the CCTV but we try to be each other’s keeper. As my colleague if

we are on duty and you make a patient’s feel uncomfortable I will call you aside and talk

to you. If not, the patient has to inform us.

I: Okay thank you very much. Is there any submission you will make for other in-charges

to ensure respectful maternal care in their unit?

R: I will say the client should be “our friend” so that they will feel at ease to be with us.

That alone ensures maternal care and some clients are stubborn they don’t listen but do

what they feel is right. As a midwife, that’s our job we should do our very best to give

them the care and shouldn’t be extreme that we abuse them.

I: In terms of privacy issues especially your ward that’s very opened (smiles).

R: Privacy (laughs). If the patients are not many and we can use the screen we will but

whereby the patients are many and can’t provide screens we should be improvised by

using the patient’s cloth to provide privacy being injection or vaginal examination.

I: Thank you very much. Do you have any other submission?

R: No there is nothing (smiles).

I: Okay thank you for your time.

R: Thank you.

IC LAB 5 30 MINUTES 19 SECONDS

I: Please as I said in-charge have you agreed to grant this interview?

R: Yes please.

I: In-charge please what’s your age?

R: Please I’m thirty-four (34) years.

I: Please what’s your educational qualification?

R: Erm… Diploma in Midwifery.

I: Okay. Please how many years have you practiced midwifery?

R: About eleven (11) years.

I: Okay. Please how many kids do you have?

R: Two (2).

I: Are you married please?

R: Yes please.

I: How do you understand respectful maternal care please?

R: To my understanding is how you treat someone when she comes to deliver…yeah.

I: Thank you. So, as you’re in-charge what have you put in place in your unit to promote

respectful maternal care during your shift?

R: When I’m on duty I make sure to do the needful so that others can learn from me.

I: Okay. When someone in labor refuses to allow you to examine her and you know what

she’s doing can put her life at risk what do you do?

R: We talk to her about the implications but if she insists we document our effort then finds

a way to resolve it.

I: Some patients behaviour is frustrating and it’s difficult to work on them maybe not you

but in your unit what strategies do you take to solve that?

R: Communicating and pampering but some patients still don’t pay heed hmm…you’ve to

scold them not frequently but to the extent of beating a patient to push I have not

witnessed that.

I: What about getting two (2) people to help you restrain her leg if you have enough staff?

R: Sometimes it’s good but some patient won’t allow when two people restrain her but

when you shout on her a bit it scares her to comply to what you’re saying.

I: When a patient does not comply and you shout on her as a strategy what do you say to

her after the delivery?

R: After delivery, I apologised to the patient and explains to her the reason I shouted.

I: As the in-charge here have you seen or received any report that a midwife has treated a

patient badly as a result of not complying to some rules?

R: I’ve seen some but wasn’t that offensive to say is a bad act.

I: Can you share that experience with me?

R: Recently was on night duty when a lady came as a referral from a facility with no

midwife and a letter and when she was asked why she explained that the midwife told

her to take the lead. Though that’s not how it’s supposed to be but we admitted her and

her behavior was uncalled for. She even entered theatre were someone was being

operated and told the doctors she wants to be operated else she’s not leaving. We got

furious and told her to do whatever until it reached the second stage and came to tell us

she’s ready.

I: At the second stage, did she comply?

R: It was not easy she even closed her leg not to allow the baby to come.

I: What did you do?

R: We did nothing until the baby came and she allowed herself then we took the baby to cut

his cord.

I: Okay. Have any patient complained about a midwife who did not seek permission from

her before starting delivery procedure?

R: Oh I’ve not received such complain.

I: Have you witnessed some yourself?

R: Yes I have but I think before you begin a delivery procedure you’ve to seek for the

patient permission before you begin.

I: For instance; if you come to work and witness such thing how do you react?

R: Oh I called the midwife and inform her how things should be done not otherwise.

I: What happens after that?

R: Some accept the wrong and apologised others too sees nothing wrong about it so I just

leave the person.

I: Do you see any improvement after the talk?

R: Oh yes I do see improvement in most of them.

I: Okay. How many privacy screens do you have here?

R: Hmm (laughs) please we’ve four (4).

I: Is the four (4) adequate?

R: The four (4) is not adequate because one (1) is for admission, the other three not enough

for the cubicles so normally we screen the staff no matter what. Some may get the

screen others won’t get.

I: How many beds are here?

R: Each cubicle has two (2) beds.

I: Okay and how many cubicles?

R: We’ve about ten (10) cubicles.

I: Alright..so how do you ensure privacy of patients as there is inadequate screens? Have

you written a request about it or maybe you’ve informed the nurse manager?

R: We do complain to our immediate in-charge but her response is that she has written to

the management so waiting for a reply.

I: Who receives the written request?

R: The business management receives the request.

I: Business management approves before you purchase it.

R: Yes please.

I: What challenges do you face in order to ensure adequate equipment as I know privacy

screens are not the only challenge in your unit? What are the main problems?

R: Main problems is the management because you’ve to wait for their approval. The

hierarchical structure of the hospital makes it necessary to wait if your in-charge

proposes a letter to the management.

I: Okay they are the problem?

R: Yes because when they provide us with the equipment we will use it to work. Even the

ones that needs to be repaired is also a different issue when complained to them.

I: In cases like this, what are the options of the in-charges?

R: We manage the few ones available.

I: Do you train your new staff or staff in your unit about the rights of a patient?

R: Yes please. At times our immediate in-charge organizes training for the whole ward and

also the new staff are trained on how to treat patients and the patients perception about

health personnels.

I: What are the response after the training? Any changes?

R: Yes we see improvements on how they work.

I; Do they take the training serious or they see it as normal?

R: Please most of them takes it serious.

I: Is the meeting mandatory when organised?

R: We organised it during shifts ( morning, afternoon and night) but the night shifts

normally joins the morning shifts in order to get all staff to participate.

I: Do you organised meetings to ensure that your staff and new staff treats patients with

respect and diginity?

R: Yes please.

I: Upon all these meetings, what are the challenges do you face? Let’s take the staff for

instance what are the challenges when such meetings are organised?

R: We get comments like you’ve been educated so when a patient comes we will call you to

show her the respect (laughs). Such comments at times makes you feel bad.

I: Aside the comments, is there any challenges in terms of resources to organize the

meetings?

R: No please, is just a normal talk no resources needed.

I: What do you do to help a patient that can not afford her medicines?

R: We don’t face such issues because our prescribed medicines can be afford with just two

Ghana cedis (2.00) and four Ghana cedis (4.00).

I: What about those who can’t pay their hospital bills?

R: (Smiled) hmm…after delivery they are taken to a different ward to be discharged so

such callenges are not faced here.

I: Do you work with social welfare here?

R: No please we don’t work with them.

I: Okay. What have you putting in place to encourage a patient to report abuse by a nurse

or a midwife?

R: So far there are no procedures in place for a patient to report an abuse but at times on

duty a patient can make a complain about how she was been treated.

I: Has anyone reported a bad behavior of a nurse towards her to you?

R: At times they make comment (the nurses on shift in the morning, afternoon, or evening

have no manners).

I: Do they tell you what other shift does?

R: Yes one can even point at a specific nurse that she scolds a lot.

I: In cases like this what do you do?

R: Please I do ask of the patients offence and tells the nurse to take it easy the next time.

I: Are all patient treated equally irrespective of her age, class, work or education in order to

voice her grievances?

R: Yes please we do treat everyone equally…but apart from screening the staff we treat

patients equally.

I: Okay. What are the measures putting in place to monitor reported abuse cases in your

absence?

R: Erm…there’s a group in this hospital from time to time I’ve forgotten their name.

I: Quality Assurance?

R: Yes they come around and chat with patients in case there is any abuse patients report to

them.

I: With this the patient does the report herself?

R; Sometimes patient relatives do the reporting or they report when they come around.

I; Have Quality Assurance given you a report or complains from patient?

R: No I’ve not received such report from them.

I: In-charge as if I asked this question but have you gotten a complain from a patient that

someone has beaten or pinch pushed her?

R: No please I’ve not gotten any complain. At this place, being beating, pinching does not

really happen but for scolding it does. Patient rather beats us.

I: They beat you? Eeii

R: Yes (laughs)

I: In situation like this, what do you do?

R: When it happens we see it as the pains the patient is going through. After delivery, we

tell her what she did instead of apologising she gives a check of it. This at times makes

you angry to reply her. Mm…at times a patient hits you with her leg.

I: In labor?

R: Yeah, others do apologise and some do not but rather reply you that is your job so her

leg hitting you is nothing. (laughs).

I: Hmm what an issue so with such a reply what..

R: When she replies is you are human (laughs) you will be angry but rather tells her that is

not how to talk at least apologised about what you did.

I: Is there any challenges we made no mentioned of that you will address/ others complain

about staffing so here when it comes to staffing ois there any issue?

R: The number of staff is not enough in case theres workload it becomes difficult to control.

We’re human so if someone can not control the pressure then scolding set in. Iwill plead

everyone is trained about the respectful maternal care. It will help.

I: Those who came have you seen any improvements?

R: Please there has been improvement about those who came.

I: The hospital as a whole what measures can they take to speed up the process when you

are in need of equipment?

R: (Laughs) if the hospital can create an account for such petty things it will help.

I: Okay. Lastly what should be put in place to help you as an in-charge to ensure respectful

maternal care?

R: Please as I stated it will be good if everyone is trained about respectful maternal care

because after the training the relationship between us and our patients has really

improved. Though not hundred percent (100%) but it is okay. And also the hospital

should be able to provide as fast as possible the inadequate equipment in order to ensure

respectful maternal care.

I: Please is there anything to address or we are done with all?

R: Yes please we are done with all

I: Okay I am grateful.

R: Same here.

IN-CHARGE 3

AUDIO LENGTH (34:46 MINUTES)

I: In-Charge, good afternoon. I am grateful to you this afternoon for agreeing to have this conversation with us. And, please, as I explained to you earlier, have you agreed to have this interview with me?

R: Yes please.

I: OK. Please, In-Charge, how old are you?

R: Thirty-seven (37).

I: Please, educational qualification?

R: Tertiary.

I: Tertiary. OK. In-Charge, please, how long has it been since you started practicing midwifery?

R: Well, twenty-three (23)?

I: 23? But you are really cute though! Indeed.

R: Well, I am not sure. I am not sure I’ve clocked 23 years yet.

I: Really?

R: From 2006 till now.

I: From 2006, then it should be about…

R: Yeah….

I: …fifteen (15) years.

R: Thirteen (13) years. Fifteen?

I: Yeah, fifteen years.

R: Really?

I: Yeah, for as long as years, I would have been surprised.

R: …would have been surprising (both burst into laughter).

I: Eiii!

R: 2006 till now….

I: Yeah. OK. And how many children do you have?

R: I’ve not given birth yet.

I: OK. And please are you married?

R: Yes.

I: In-charge, please, when we talk about respectful maternal care or maternity care, what is your understanding of the concept? Can you share your thoughts on this with us?

R: Yes. My understanding is that, any woman who comes to deliver, or who is pregnant and is here at the hospital, you must treat her like we treat human beings, kind of.

I: Uh huh….

R: Uh huh…so as I want to be treated as a woman, I should treat all other clients too as such. And, pertaining to that, respecting their views and meeting their needs as individuals.

I: OK. Thank you very much. Well, In-Charge, right now, you are the one in charge of the labor ward. So when we talk about respectful maternal care, in your opinion, what is your responsibility in this?

R: OK. In that regard, what I do is that I make sure I lead by example. Therefore, those patients that….though I am the in-charge, I don’t always sit at the table: I do work. When you get in here, I work and mingle with them like…uh huh….

I: Yeah.

R: So, what I do, I know juniors (staff), they look up to seniors to copy. So what I do, they look up to me to copy from me. Thus, I make sure that I am treating the patient with respect so that the junior staff who just started working, if she observes what even the in-charge is doing, she too can copy and do likewise. And from time to time too, I remind them of being conscious of how they deal with these mothers.

I: Uh huh…so, In-Charge, do you have anything else you might want to add to what you’ve already said about your responsibilities on respectful maternal care?

R: Well, I also saw it that, because I was not directly involved in the study and those who were initially trained, I advocated that more of them (staff?) can go for the training. Thus, I believe it was last year…

I: Mmmmmm….

R: ….they brought some people to be trained more.

I: Mmmmmm….

R: And those who went, I made sure that when they came back, they disseminated the information to others, and gradually, I think care is being improved.

I: OK. In-Charge, what strategies do you adopt to help women in labor whose actions may put baby and themselves at risk either directly or indirectly? So, let me take it that the women who come here to deliver, for instance, there are people who refuse to push when they are told to. Do this….whatever it is that you need her to do to aid her own self, when you do instruct her, she will ignore you. In such instances, what are some of the strategies that you use to help those women?

R: Well, most of the time, what we do is to encourage them to do the right thing, and letting them know the consequences of their behaviors. There are those who do not know that maybe ‘push’, and she is not pushing, probably, baby will come out asphyxiated or anything of the sort. But if you explain things to her….

I: Please, what do you mean by asphyxiated?

R: Well, by the time the baby comes out, it might not be able to cry, it might be extremely weak, it can’t….like any other baby does, you know?

I: Mmmmmmm….

R: Then, after you’ve talked to her about these, you will realize she will start putting in effort to do what is expected of her. Sometimes too, personally, I believe it is the tone you use to speak to them. Thus, just telling it in a nice way, because that is what is required. In telling it in a nice way, they usually fall into play and go by it.

I: Mmmmm…and have you had situations in which you may have to use, let me say some extreme….

R: Force?

I: Uh huh…maybe some force or….

R: OK, for this place…when it comes to this ward, maybe it may have happened behind my back, but since I am…since I’ve been here, if I am on duty, we’ve had instances when the delivery couch is there, the client says she is going to deliver on the floor.

I: Mmmmmmmm…..

R: You have to do it. And that is why I am saying maybe when I am not around, something else happens. But for the kind of people I deal with, at least, I know taught them…Aha. But to beat a client, or something of the sort, personally? No. Uh huh. It might happen, and I will not say that…I will not deny it (deny the possibility of it happening behind my back) but I’ve never observed anything like that yet.

I: Uh huh…you’ve not observed it personally, but have you heard of the sort before? It might not be physically beating someone but sometimes you may observe it was inappropriate the way a client was spoken to….

R: Abusive words, yeah?

I: ….and even after the client is delivered, she may approach you and say ‘In-Charge, the way this particular nurse spoke to me, I am not happy about it’.

R: Oh OK.

I: So, it is not just about being beaten or things akin to that….but also in the way people speak to clients.

R: Oh OK, in talking to clients and such….

I: Yeah.

R: In that regard, the truth is that most of the time, the patients themselves do not normally come to complain, but it is the relatives who will come and maybe complain about one or two staff.

I: Oh OK.

R: And then when you see this, you will also call them (staff), and they will also come and tell their side of the story. But, always, we say that ‘the patient is right’.

I: Mmmmmm….

R: So, we have to thrash (out) issues and I’ve had instances I’ve had to go personally and apologize on their (staff) behalf.

I: Yeah. Oh OK.

R: Because, the staff feels that if she had not done what she did, there is the possibility that the client would have lost the baby. And the client also feels that …. Well, the patient themselves are afraid coming to you, but once they tell their relatives, the relatives will come to you, because they didn’t have a direct contact with you. That is it.

I: Oh OK. So when incidents like these happen, you do apologize?

R: Yes, I’ve done that severally.

I: OK. And then, have you seen or heard, or have you received reports that a healthcare provider – either a nurse or a midwife – treated a woman roughly? Like, push, beat, slap, pinch or physically restrained, or gagged them (clients) while delivering on the ward?

R: Well, I have heard (of such incidents) ,and I am saying that, when it comes to physically seeing such things….

I: Mmmmmm….

R: And that is why I said it, if it does happen behind my back, I do not know. But I have heard, even here and other places, but as to….

I: But not in this ward?

R: ….and as I keep saying, for this ward, no information has reached me yet to that effect.

I: OK.

R: No patient has yet come to me to talk about this but most of the time, it is the words: the way she spoke to me, I did not appreciate it. Uh huh…..

I: Mmmmmm….Mmmmmmm….

R: …..but not ‘She beat me’, ‘She pinched me’, and things of the sort.

I: OK. And when it comes to gagging them, what is that one? The one in which…. (talking at the same time)…

R: The one in which….pinching their….OK.

I: And for that….

R: No. For here, what I know is that, you will hear someone say that ‘Madam, if you don’t push, your baby is getting its breath cut. Do you want us to transfer your baby to MBU?’ Or sometimes if they are not pushing and we are advocating for vacuum delivery, then she (midwife) might say ‘Madam, if you don’t push and we use the machine to extract your baby, the maybe the head…when it grows up, it might be dumb (not intelligent in class)’…

I: Mmmmm….Mmmmmm….

R: …which is…when she was done with the delivery, I personally brought her into the room and told her that it is not the indication for, or the consequences for vacuum delivery. You have to….

I: You brought who into the room? Is it the one….

R: I mean one who said it.

I: Oh OK, the midwife.

R: Actually, it wasn’t the attending midwife. It was a student – a rotation – the one who was standing by. I: Oh OK.

R: It seems everyone around was eager to help the mother to bring the baby out…

I: Yeah.

R: … but we say something so you can know your consequences: what can befall you if you don’t move on or something. So, it is not …. I don’t know.

I: Mmmmmmm….so you’ve not encountered anything like that here yet, right?

R: No.

I: OK.

R: And you know that when superiors are around, the behavior of juniors is different (chuckling)…

I: Well, that is true….

R: You understand?

I: Yeah.

R: That is why I told you that it could be that it happen, but if it happens, it is behind my back.

I: Mmmmmm….

R: And it hasn’t been brought to my notice yet, because if it comes to my notice, definitely, I will have to deal with it. Uh huh…

I: And then, have you seen or heard, or have you received a report that a midwife treated a woman without seeking her consent or attend to them without permission when delivering at the ward?

R: Seeking consent as in their? For example, you having to tell that maybe ‘Madam, I am going to do this procedure for you’?

I: Yes. For example, when a staff is about to do a vaginal examination to determine if the cervix is opened, but let’s say she just comes without any explanation and summarily goes to ‘Madam, open your legs’. Or….

R: Oh OK! OK.

I: … when she is about to give a client an injection, the moment she gets to the client ‘Madam, turn around’.

R: Oh no….in this place? No.

I: Nobody has reported any such thing?

R: I think that we’ve gone beyond such points (laughing) because mostly, you see, mostly, consciously, everybody…I don’t know. When any one on this ward is going to examine a patient, ‘Madam, please, I am here to find out if your cervix is opened’. Sometimes, they even call you –the mothers themselves call you that ‘Please, come and examine this for me’.

I: Mmmmmm….

R: So when you get there, you also say that ‘Please, lay down properly and let me take a look’. So when the midwife is done, she might say ‘It is left with a few inches’ because I believe in just seeking consent and giving feedback, which for right here, I will give them a plus. Even the doctors do it.

I: OK. And then, In-Charge, how many screens do you have here, in your ward?

R: We have five (5).

I: Five, and are they adequate for you?

R: Given the choice, every cubicle should have a screen, but….

I: Mmmmmm….how many are your cubicles?

R: The cubicles are ten (10).

I: Oh Ok.

R: But most of the time, we use the five: one for where we do the charging…

I: Oh OK.

R: You remember when you first entered, when you first enter the place, where we receive the people?

I: Mmmmmm….

R: So that when someone is sitting around: you know that if there are more than one patient, the other is waiting to be seen while the first one is being attended to?

I: Mmmmmmm….

R: So the one waiting might not see what is happening. Thus, in that area, every time, one of the screens is placed there. Then we rotate the four between the cubicles.

I: The ten cubicles….

R: Yes. But we encourage them (clients) to cover themselves so that when someone is passing by….we wanted to do this as the second stage, where the curtains are….

I: Ah…the second stage, I’ve seen that there are curtains in that area.

R: Aha! But here, it was a difficult decision here. I think it is a managerial something (issue)….when they were probing….this costed a little. Uh huh…thus….

I: When they did the second stage?

R: Uh huh…thus, requesting for another one? So, what we were provided were the screens. So we use them as and when the procedures are being done.

I: Well, but you were saying that, right now, those are the ones you are rotating. And then you also said that when they came to renovate the second stage, they were saying that the cost was too expensive and all that, so looking at the fact that what you do have is adequate, what have you done about it? Have you requested for any?

R: Yes. I have requested for more. We’ve requested for more, but initially, they were three (3) and some got out of shape. So when we requested for more, we got a few added, and now we have the five. So……therefore at least, I hope that with time, we will get more. But what we do is what I am telling you: when the procedure is being done, we will screen so that … well, when you enter, there are two (2) people in a cubicle…

I: Yeah.

R: Aha…so that when you are taking care of one, the other one should not see what we are doing.

I: Mmmmmm….

R: So that is when we screen and….

I: Oh OK.

R: But after that, we encourage them (patients) to cover themselves so that when someone is passing by, the people may not see their nakedness.

I: Uh huh…..so with the screens, when you requested them, like, you requested for who? Which unit do you report to?

R: Thank you. My superior…my boss…my next manager, then it will pass through the business manager, then they will also send it to the procurement, or something like that.

I: To the procurement, OK.

R: About it, eventually.

I: Well, when it comes to the screens and the curtains you were talking about, I know you’ve talked about some of the challenges, but can you throw more light on it? What are some of the challenges that you face in ensuring that you have adequate curtains and the screens?

R: Well, just as I was narrating to you, the department is a very bid department and when you put in your request, they prioritize…they see which one is like more pressing…

I: More important….

R: ….than the others. So maybe, when you go there, something might be more urgent in another unit. Probably, if they tell you to manage with what you have, you have no other choice than to manage. But as and when…..well, even when donations come in, they see us first – the labor ward….

I: Oh OK.

R: When it comes to that (the preferential treatment of the labor ward), I won’t deny it. They see us first before they give to other places.

I: Mmmmmm….

R: Uh huh…unless we are not in need of these items. But, gradually, if you push and push, it will eventually come. So I am still pushing.

I: OK. But do you get frustrated sometimes? That you’ve written the request and put it in and you will follow up intermittently, assiduously, and all?

R: That is a fact, but as I’ve been saying, (smiling) because we don’t have the power to go and buy it ourselves, you just have to wait for them (management?) to bring it.

I: In-Charge, right now, let us go to your staffs. Here in the labor ward, do you have any in-service training that takes your staff through the rights of the child-bearing woman.

R: No. We do not have any scheduled or laid down trainings like that. But personally, what I do is, at the end of any performance appraisal, I see where the shortcomings are, and I make it a general on-the-job training for them. That is what I do. But we do not have a specific…especially with the rights of the child? No.

I: Mmmmmm…..

R: It is those who went….

I: And for that (rights of the child?), have you ever had an in-house training for them?

R: Those who went for the training, they are those who, when they came back, they were encouraged to disseminate the information (to the others), which we did. And, those, they were given soft copies….ahaa….which I know they shared out there, I encouraged them to share. Some were put on our platforms – the ward platforms.

I: Oh OK.

R: For you to read…so….

I: OK. So after the training, what is your overall impression about, let me say, how they are applying what they learnt on the unit?

R: OK. I would say that I have not been with them for a very long time…

I: Oh OK.

R: …but from the time I came to the time the training started, I would say that things are much better.

I: Mmmmmm….

R: Things are much better, because we have trainer of trainees here….

I: OK.

R: The one you met outside? The one with the short cut hair?

I: Oh OK.

R: She is a trainer of trainees.

I: Oh OK.

R: Ahaaa…and, she is also a shift in-charge who also leads by example.

I: Oh OK.

R: And that is why I keep saying that when the junior staff sees the senior doing something, how dare you….

I: She will also emulate…

R: Aha! So I believe…..

I: OK. And then, do you have, aside what they went to, do you have any other in-service training that takes the staff through that ensures the child-bearing women are respected and treated with dignity?

R: Apart from what your group did, we do not have any other resources like that. We’ve not yet gotten any such invited again. So this is the first of its kind that has come up.

I: OK. Well, when you consider the training of your staff, what are some of the challenges you do encounter in training them and the new midwives?

R: Well….I would not say that it is a challenge we cannot overcome, just that, getting everybody at the same time is the issue. So what we do is the usage of the main platform, then sometimes we have virtual presentations. So I’ve given the mandate to one person – one of the trainers – she does virtual presentations. Like…she does recorded presentations and she puts them on the page. And when the students too…the rotation and the other students come, as part of their rotation, we do also mention it that for here, patients do come first. So anything you do not understand, ask your in-charge or ask before you do something.

I: OK. And then when do you do when it is reported to you that some new mothers cannot pay for services?

R: Eh…..for this place…

I: For the labor ward….

R: For the labor ward, we don’t deal with payment of services.

I: OK.

R: Especially when we are going digital now. Ours is to render the services, after the fourth stage monitoring, they are sent to the lining board, that is where the payment and all the other processes starts. So we really, really do not encounter such problems here.

I: Oh OK.

R: Because it is there that they would be discharged and the bill would be calculated, and then it might be discovered that the client would not be able to pay…..

I: Ah…when they go to SPONT? Or that place is what?

R: SPONT, yes.

I: OK, SPONT.

R: Yeah, at that place. So here, I wouldn’t know much to talk about.

I: OK. So you don’t work with the social welfare department?

R: No. No, no.

I: OK.

R: Because you will finish rendering the service before you find out the client cannot pay. Because this place is always an emergency; we don’t wait for money before you do anything. The only thing that you can anticipate is that you may realize she came with very few things or she brought absolutely nothing. That is the only time you may be able to determine that she may have problems when discharged (indicator) or something of the sort. But we don’t directly deal with them. It is after discharge that we involve the social welfare people.

I: OK. And with that, let’s say the client comes with nothing when she is coming, in such a situation, what do you do?

R: Most of the time, we use what they have. This morning, we had an instance. When the mother came – it was an emergency – and we had to prepare her for section, but she brought nothing.

I: Uh huh…..

R: So what we did was, we begged the theatre staff, the cloth the lady was wearing, she gave us the consent to tear it into pieces to use it as cord sheets. And we decided it….

I: Oh OK.

R: Till….by the time her items came in, she was already inside; she had entered the theatre. Then…but they had already used the theatre first towel to receive the baby, but how the rooming-in nurse will wrap the baby, keep the baby warm was the issue. Because they had already used the cloth we split into pieces, so when her stuff arrived, we just sent them in to the staff.

I: OK. But In-Charge, in your unit or the hospital as a whole, what reporting systems are available to encourage women who come to deliver here to report abuse? For example, if someone is here and she feels like she wasn’t treated good, or she had some challenges, what reporting systems are in place here?

R: What I know is that we have the QA, the QA department.

I: The Q? Quality?

R: Quality Assurance, uh huh….

I: OK.

R: And they have reps on every ward. And so those are the people who take up such issues.

I: OK.

R: And where our nurse manager’s office is, it is an open place and it is also an easy-to-find location. So most of the time, they report to the in-charges and we cannot deal with it, we send it to her (nurse manager) for her to take it up. Yeah.

I: OK. Well, since you’ve been here, have you ever had a report from someone who came for a delivery? Let’s say the person approached you directly to tell you that maybe the midwife or the staff who were on duty abused her?

R: Well, that is what I initially told you that personally, nobody has ever approached me in that manner. Whether it has to deal with physical abuse or other abuses, but it is the relatives who …. You might be just passing by, it is not even the case where the relative intentionally comes up to you…you know they sit at the reserved place outside, so she might be sitting outside at that place….

I: Outside there, ah…..

R: Uh huh….and you might hear someone having a conversation about an incident. For me, if I hear anything like that, I will get closer and ask you ‘Oh what happened?’ and by the time the person explains everything to you, then….initially, you don’t have to wait for the other (person’s viewpoint?) because if it touches one of us, it touches all of us. So you just have to apologize on the person’s behalf. Then, you come inside to also listen to what really happened so that what went wrong, you can correct, and what they didn’t do and that you have an idea on, you chip it in so that next time, they don’t repeat it.

I: OK. And please, when it comes to the system, when you explained that we do have quality assurance, and some people are also able to go to the nurse manager, from your perspective, how friendly are these strategies to all women? For example, those who are educated, those who are not educated, those who are rich, those who are not rich…..

R: Oh…from what I know or what is happening around, once you enter that place, for KATH, our patients come first. So once you enter the place and bring your problems, they don’t look at where you are coming from or what you can provide or not. They just take the matter up, and investigations are done to find out what really happened. Then, as I said, if sanctions are needed, they do sanction. If not, they find a way to put things in the right way.

I: So it means the systems treat everybody equally.

R: That is what I know….

I: Mmmmmm….

R: Unless it is otherwise, I don’t know. But that is what I know.

I: OK. And aside the quality assurance and going to the nurse manager, do you have any other system or what else have you put in place in the view that you can use such a system to monitor occurrences of mistreatment even in the absence of received reports. So, let’s say that even the woman did not come to you to report what is going on, or a relative never came to report what went on, what systems are in place to tract abuse?

R: We don’t have such systems in place. Since….in other places that you go to, you may have CCTV cameras to at least view what happened, but here, we don’t have such things. We don’t have anything like that.

I: OK. And in such instance like this, what are some strategies that you think may have helped?

R: Hmmmm….like I was saying, if you really want to monitor things well, then you really needed maybe a CCTV camera to know…monitor what really happened. You may not hear what is being said though (soundless?) but if someone is abusing someone, you will know. Maybe her body language, expression…it will make you know. But apart from that, well….I really don’t know.

I: Mmmmmm….and apart from the things we’ve touched, what are some other strategies that you think could help?

R: Erm….strategies like?

I: Those that can help someone who has come here to have a baby. Remember I was asking you that sometimes someone might beat her, or pinch her nose, or something like that. So in such an instance, what are some of the strategies that, in your opinion, could help the mothers to comply? Maybe if you tell her to push, she might push so that everything will proceed smoothly so that nobody will talk anyhow to the mothers?

R: OK. Well, I think that in all these things, they are already in the system because when the mothers … for the labor ward, we only have a few hours’ encounter with the patients.

I: Oh OK.

R: You see? So depending on the patient’s stay with you, (it is only in) rare cases that the patient spends twenty-four (24) hours with you. But what I know is that, before they come to the labor ward, certain things should come into play. She’s been to the ANC, what was she taught in ANC? Because, telling her what to expect in labor, if she is experiencing those things or she is seeing those things, she would not be too alarmed, be too stressed out, because at least she knows this is supposed to happen. And mothers react the way they do because of the fear of the unknown.

I: Mmmmmm…..

R: You know? So I think that most of our focus, and that (those) ANC visits….it is a very long time. And so they bond more with the staffs there, and so, if you are my friend and you tell me something, I listen to you more other than meeting someone for the first time in pain…

I: Mmmmmm…..

R: …and then not knowing even what I am going to experience in the next one hour or two hours. So, in my view, what I think is right is what I am doing as a patient. Therefore, probably, we should intensify the kind of education we give to our mothers at the ANC. Or, even before preconception (before conception?). The preconception care that we give to them, I think some of these things should be…like…concentrated there, so that they only see the labor ward as a passing-through stage, you know? So that if you go to a place fully prepared, you will face the system as it is, and everything goes as planned.

I: Mmmmmm….

R: That is personally my views. Yes.

I: Please, do you have anything else you may want to add?

R: And….I will also say that, sometimes, as human as we are, we need constant – how do I put it? – Reminders. I encourage one of my staff to get posters around so that when I am working as a midwife, and I look up, I can remember. That patient I am working on needs to be treated respectfully, and it is a project work, so she is still on it. She is doing it.

I: Oh OK.

R: Uh huh…of which I believe that maybe next year, by the time you come back for another follow-up, you will see those things around. So that patients will know their rights, the midwife will also know her right, and her duties as well. Just a constant something: just to keep people….

I: Uh huh…reminded.

R: Uh huh…because as humans, we are not the same: different people have different ways of dealing with issues and such. But if it is a constant something, you will look at it every day, it keeps ringing in your mind and you do accordingly.

I: OK. Please….

R: Thus, with the issue of screens and such, if at the end of collecting your data, and then, especially with the issue of privacy, and you have the opportunity to help us, then please come and help us. Then you come and help us (laughing and the interviewer joining in the laughter).

I: Please, is there anything else? Anything you might want to add?

R: Please no. But what I would like to say is that we thank you very much…you and your team for giving us insight into some of the things we do which, we thought probably were right. Actually….because when we came for the training, there were issues when raised and you were sitting there listening, you will tell yourself ‘Ah? But I thought I was helping’.

I: Them…..yeah…yeah.

R: You understand?

I: Mmmmmm….Mmmmmmm…..

R: When some brings your mind to remembrance, you the perpetrator didn’t know you were doing the wrong thing…so when someone prompts you to the issues, it helps. Thus, we will thank your team, you and your team members for the fact that such a program has been rolled out. And I believe that, as at now, it is being thought in the schools. So when by the time the juniors come here from the schools, it would be already inculcated in them….

I: Yeah…it would be inculcated in them already.

R: Therefore, when such a person is performing the needed things, they will not be something new.

I: Mmmmmm…..Mmmmmm…..

R: Uh huh…thus, we do thank you.

I: Please, we also thank you very much.

IN-CHARGE 6

AUDIO LENGTH (39:24 MINUTES)

I: In-Charge, good afternoon.

R: Good afternoon.

I: I hope you are doing well?

R: I am doing fine.

I:Uh huh….In-Charge, just as I explained the main objectives of this research to you, have you agreed to participate in this interview?

R: Yes. Yes.

I: OK. In-Charge, please, may I know your age?

R: I am forty-four (44) years.

I: OK. Educational qualification?

R: BsC.

I: Ah…so, tertiary.

R: Tertiary.

I: Please, for how long have you been practicing midwifery?

R: How long?

I: Uh huh?

R: Actually, I am a nurse-midwife…

I: Oh OK.

R: …so I’ve practiced, in all, midwifery/nursing, eighteen (18) years now.

I: OK. Please, what is your parity status now? How many children do you have?

R: I have two (2) children.

I: Two children. Please, are you married?

R: Yes.

I: Oh OK.

R: I am happily married (both burst into laughter).

I: We thank God for that…uh huh. OK, yeah…In-Charge, when we talk about respectful maternal care, what is your understanding of that term Respectful Maternal Care?

R: Actually, respectful maternal care, in my own understanding, is a kind of care that you render to mothers that are in need of a kind of care that is actually…basically, it is in maternal cases, mostly you see it in Obs and Gynae …Uh huh…a kind of care that you give to them that will help them in a kind of condition or a situation. So, it could be during their gestational age – maybe during their pregnancy time or duration - or it could be as a result of gynaecological case – in which maybe they are not feeling well or they are very sick – and then you render them the care according to their need. I mean, and at the end, you put a smile on their faces.

I: OK, so In-Charge, as the deputy in-charge of this place, from your understanding, what role do you play to promote respectful maternal care in this unit?

R: Actually, I don’t work here alone – I work with my colleagues.

I: OK.

R: Some are of the same rank and some are junior in the profession. Mostly, they see me portray the patient first; the patient’s interest first, and what the patient wants. And personally, personally, I like allowing the patient … I care for the patient like a royal ‘someone’ because the person actually trusts me: that’s why she decides to come to me for the help or the care. So on the ward, I tell my colleagues that we should portray their interests first and care for them as in if a patient is in labor, at times they are psychologically disturbed, you should have that at the back of your mind, you don’t shout at the patient when they start misbehaving or playing something in the wrong direction, you have to put them right and you must have…be ready to pull the long rope with them in the sense that the situation in which they are in is for the meantime: immediately they deliver, everything goes back to normal. So we portray their interests first and allow them to feel comfortable, to feel at home, and then cooperate with our treatment.

I: OK. So, In-Charge, on the unit, on this unit, what are some of the strategies you use to help women who are in labor whose actions may put their baby or themselves at risk? So for instance, maybe a patient comes in and the patient is in labor, and maybe you want to perform vaginal examinations and then the patient is not willing to open the legs for you to have access and perform that examination. So, in such an instance, what are some of the strategies that you use to help these women?

R: Actually, we do communicate with the patient. We do communicate and re-communicate (both smiling) because at times, they don’t actually understand why you are doing….especially like the teenagers – a teenager that is pregnant and is about, almost getting to second stage. You know the pain is so high, and in Africa, the strategies to relieve pain is so expensive, so, we tend to communicate and re-communicate to them that if you don’t let us know what is happening to you, we won’t be able to help you very well. So let us assess your dilatation, let’s see what is happening, so that we can take the appropriate steps at the right time.

I: Mmmmmm….

R: Uh huh…and at the same time, we make sure we give them privacy if they are….when they are in labor and …. There is a place where we admit them; we don’t admit them amidst the crowd. We take them a bit far away from the other patients that are on the ward so that they can feel comfortable, and then allow us. So we make use….when they are more than…because here, we have only one delivery bed, and we have one examination bed, and at times, if the area is occupied and I need to assess a woman’s dilatation, then we make use of the screens while they are still in their beds so as to help the patient.

I: OK. In-Charge, have you seen or have you heard or have you seen a report that maybe for instance, one of your midwives have treated a patient badly, like maybe pushing the patient, beating, slapping, pinching, physically restraining the patient…have you received any such report?

R: Mmmmmm….so far, no. So far, no…because the clients know us well on the ward, especially maybe the deputy in-charge or the in-charge in which (to whom?) they can complain: no patient has lodged any complaint of that sort.

I: Oh OK.

R: Yes. So far, I think the midwives on the ward have been trying their best. And the….they allow, they give the women the respect. To some extent, they give them the respect. You know at times, maybe, psychologically, the caregiver too might (smiling) also be a bit disturbed, maybe by speaking (in) a louder voice..I mean word, or something. Or, maybe it might happen, but no client has ever complained of any maltreatment.

I: Mmmmmm….but has there been an instance where maybe you witnessed a midwife shouting or verbally abusing a patient?

R: Yes. I have witnessed one: maybe more than once. And I actually corrected it, and the fellow is a junior colleague. Uh huh….so at times, I think that fellow…it is a junior colleague, so we had to caution the fellow and tell the midwives that that is not the way to take care of them (patients). That they should actually give them the chance and allow them because at times, patients/clients have different pain thresholds; so you in a situation whereby they can’t (control the pain), you just have to allpw them express themselves.

I: Mmmmmm….

R: But shouting at them will rather create more chaos than allowing you to take care of the patient well, and it has actually corrected issues on the ward.

I: OK. So the example you just gave, what really happened? Can you throw more light on it?

R: By?

I: Yes, you were saying that….

R: You don’t shout at the client…

I: Yes, like what necessitated that response from the nurse?

R: What was the response of the nurse?

I: No. What actually triggered the response from the nurse? The example that you just gave….

R: Oh, what triggered was….Mmmmmm….the midwife was scared that the woman couldn’t hold herself or contain the pain she was passing through, so…..

I: She was in labor….

R: Yes, in labor, and instead of stabilizing herself to push the baby out, she was rather trying to get out of bed and the midwife too was also scared of the situation whereby the baby might fall down may become a legal case…

I: Mmmmmm…..Yeah.

R: ….and it will be on her because they would…people would, I mean, and your in-charge may actually think you didn’t handle the patient well. So in that situation, you can really see that we need pain control in labor to….especially for instance, the teenagers.

I: Mmmmmm…..

R: Uh huh…they don’t know what is happening, they don’t know anything about pregnancy, it is their first time passing through it, and when the pain is coming like that, I mean comes up like that, they don’t know what to do. They just feel like ‘should I get out of bed? Should I do something by myself?’

I: Mmmmmm…..

R: Uh huh….meanwhile, you are supposed to be in bed to be helped.

I: Mmmmmm…..

R: Uh huh….meanwhile, you are supposed to be in bed to be helped. Aha….things like that. So it turned the midwife too to raise her voice ‘Please! Lay in bed! Don’t that! Don’t get down!’ Not abusive, but on a raised voice.

I: Mmmmmm….

R: ‘Lay in bed! No! No! Don’t do that!’ So that things will go on well. But no one has beaten anybody or pinched….no, no, no…..

I: (Laughing).

R: Just a raised voice to explain.

I: In-Charge, have you seen or have you received any report that one of your midwives treated a woman or maybe performed a procedure for a patient without seeking consent from the patient?

R: Mmmmmm….no. No, because, mostly, when the woman is in labor and then…probably the maternal effort is not very good or you assess the patient and she has a very big baby and then the vulva is tight somehow and the baby is not….I mean, it is actually leading to (a) prolonged second stage, you have to give, I mean, take a ….assess everything and give the right treatment to the patient to prevent asphyxia, to prevent prolonged second stage, and so on. So in that situation, you quickly tell the woman that ‘we are going to give you an episiotomy’ and most of the time, I mean, we give a local anesthesia on that side so that we quickly give so that we can bring out the baby. And after that, since the patient is already aware that we gave an episiotomy, and that the baby is out, everything is fine, then definitely, we have to repair the episiotomy for you to be well, we give some subsequent (13:48). So patients, mostly they actually….they agree to our decisions. But some that have experienced episiotomy before –the painful repair – ahaaa, they will tell you they don’t want the repair.

I: Oh OK.

R: Uh huh….we had a patient like that before, to the extent that the patient decided that we should not repair it, because the former one she had was very painful and she is not ready to pass through (that kind of pain) any more. So, was it last month? Yes. She went to bed. And we had to keep on re-counseling and re-counseling that we need to repair it. If you don’t repair it, these are the things that can happen – easy access of infection to your system and subsequent deliveries, it can extend and become bigger and so on.

I: Mmmmmm….

R: So later on, she accepted and so gave us the consent to repair and we repaired.

I: In-Charge, on this unit, how many privacy screens do you have?

R: Privacy what?

I: Screens.

R: Screens…we have up to four (4) screens.

I: Do you think they are adequate?

R: Not….about three (3) are adequate. Really two (2) are not adequate, in the sense that, how do I put it? But the available ones that are adequate, we make use of them.

I: Mmmmmm….but….

R: Uh huh….but the inadequate ones, we use them for minor procedures….

I: Oh OK.

R: ….such as (15:44) passing referrals, IV lines, and then wound dressing….

I: But then, initially, you said you had three which are adequate. Looking at the number of patients on the ward, do you think those screens are adequate for each of the patients?

R: Mmmmmm….for eah….it is not …

I: For each patient?

R: On this ward, it is not mainly labor cases that we admit. So at times, we nurse the client till they deliver. But it is, mostly, some of them…this is a high-dependency ward that has to deal with pre-eclamptic and eclamptic cases….

I: Mmmmmm….

R: And then, before you can diagnose a pre-eclamptic case, that will be around twenty (20) weeks and up. So mostly they are not term to deliver, and if they will deliver at all at that stage, it will be by CS. Ahaaa…so the labor cases that set in will be maybe referral cases or a few that are term or that we induced….ahaaa…to give birth. So it is not all the time..it is not a fully 100% maybe labor cases that we admit here.

I: Mmmmm…OK. OK, and then in addition to that, how do you ensure that the ward has adequate privacy screens? Because we know that ideally, you know, we should ensure privacy for each patient. And then initially, you said you have, I think, three (3) functional screens, and so, now how are you ensuring that we have adequate privacy screens to protect the privacy of the women who come here?

R: The few that we have, we use them on the ward, and then those, in case we have more than three (3) cases, then we take the remaining ones to the labor ward to assess them. That is how we manage them.

I: Mmmmmm….have you made any requisition to…..

R: Yeah, we’ve made requisitions, we’ve actually asked for more…and even in the labor ward, we’ve actually asked them to renovate and put screens. I mean the screens we can easily adjust to create privacy. So, almost a year now, but we are yet to hear from management. Yes.

I: OK. So, you know, sometimes it takes such a long time, so what are some of the frustrations that you have in getting these equipment which is usually delayed?

R: Actually, this, according to them, they said management will have to give the order for the money to be used. And as a teaching hospital, you know, before anything can be purchased, it has to pass through stages…uh huh. So maybe at a point, it becomes static because (smiling)….maybe they don’t see the need. I don’t know. I don’t know. It becomes static: you have to do a follow up and so on. But still, even at the recent meeting we had, I restated that we need curtains on the ward because of eclamptic cases. At times, the light - the sunlight – triggers their seizures, because they are photophobic….

I: Oh OK.

R: Uh huh….so, all these have been requested, and it is part of privacy.

I: Mmmmm….Yes.

R: Uh huh….it is part of privacy and they don’t really answer us. And so, currently, we are trying to take it upon ourselves to see if a group of people or somebody – somebody somewhere - can adopt the ward and help us on our essential needs such as this. So, that is what we are working on now.

I: Oh, OK. So, and then, In-Charge, for your new nurses and also for the old staffs, do you have any in-service training that trains these midwives on the rights of the patients who come here?

R: Yes. We do. Yes. Actually, we have a platform…..we have a platform on the ward….

I: Oh OK.

R: OK. So we make use of our platform and we post our ward guidelines and rules.

I: In-service training?

R: Yeah, the in-service training (giggling), we organize ‘mini’ in-service training for ourselves.

I: Oh OK.

R: We make use of our platforms, but we have a platform on the ward where everybody is included and then we post essential things; guidelines, how to care for our clients, how to portray their interests first, and then to give them time to be ready, to always have time to be ready to take care of them. And also, never to feel inferior to ask anyone that knows maybe more about a procedure more than you, and that we are all learning and we should always have the courage to ask people in case we don’t understand anything. So we learn a lot and we post a lot of videos on the platform for people to learn and people to see what is going on. And another thing is that we do presentations. With the help of my in-charge on the ward, we brought up the idea of the use of presentations. Different kinds of presentations….we do it (them); episiotomy repair, how to receive patients on the ward, and how to take care of pre-eclamptic cases and the use of MagSuf protocols, and how to resuscitate asphyxiated babies and how to take deliveries and then…and so many other things around our professional responsibilities. So, it is still on-going.

I: Oh OK. But…..

R: We do it on the ward.

I: OK. And so, since you started organizing these in-service training, what is your impression of the midwife response to the midwife training and are they applying it in their daily duties on the ward? What is your overall impression about the impact of this in-service training?

R: Well, actually, it has corrected a lot of lapses, and then there are some that….some embrace the new ideas and you know, change, to make a change, is not a day job. Actually, it is a gradual process, and some have embraced it, and some … maybe those that were not around especially during the live presentations, they couldn’t attend it…ahaaaa…they are yet to actually get in, to know what went on. So some have embraced it and they are practicing it and there is a change, and some are yet to.

I: OK. And then, as part of this in-service training, do you also take your staffs through how to ensure that women who come here to deliver are respected and also treated with dignity?

R: Whether I take my staffs?

I: Yes, through presentations on how to ensure that women who come here are treated with respect and then dignity.

R: Yes. Yes, we do.

I: You do?

R: Yeah, and we enforce it too.

I: Mmmmmmm…..and since you started organizing this in-service training, what are some of the challenges?

R: Actually the challenge (giggles), the challenges are interest.

I: Mmmmmm….

R: Some are not just interested (laughing) in the presentations, and some are interested. And so, it is something we should be all be interested (in) because the way we run shifts on the ward, whether you like it or not, at a point, it will be your turn to take care of someone on the ward. So if you are not in….. if you are an uninterested person or group that comes to play in this situation, there will be a problem. So the uninterested ones actually will tell you ‘I am sick, I am not feeling too well, I don’t think I can come for this presentation’ and then ‘I am off, I did night, I am tired, I can’t come for this presentation’. Anyway, the interested people are more than those that are not interested. So, I think they are still pushing it and managing (laughing, and interviewer joining in the laughter).

I: OK. And then, In-Charge, what do you do when it is reported to you that some mothers, especially those who have delivered, cannot pay their hospital bills?

R: There are a lot (giggling), especially the teenagers and also mothers that don’t have a very good home or became pregnant out of wedlock, you see? Things of that sort. So they come. What we do, we just follow the hospital protocol. The hospital protocol is: you will explain to the woman, and you will hand it over to the social welfare group. And they will handle it according to how it is supposed to be managed. So, actually, bills and moneys are not supposed to be managed by nurses. So we hand them over to the appropriate quarters.

I: Mmmmmm…..and then, and so, in working with the social welfare department, what are some of the challenges you encounter?

R: The problem is, we actually want the client to pay half or almost half of their bill before they can render help. Not just coming in to pay the whole bill for the patient to go, then it looks as if the hospital has some money somewhere paying their bills, and so they can always do anything they like (laughing). So, they are supposed to pay half of the bill, and then they can render help.

I: What if maybe the said patient does not have the financial abilities to settle fifty percent (50%) of the bill, what happens? The patient cannot still pay, you know, 50% of the bill?

R: Hmmmm…..if they can’t still pay 50% of the hospital bill, then the accountant and the business manager is….they are also involved, and they see to it. At that moment, they actually allow them to leave the ward because, most at that time, at that time, after two weeks, three weeks ongoing, they are just occupying the hospital bed. Meanwhile, other clients are still coming through and they want a place to be nursed….

I: Mmmmmm…..

R: So, they tend to put them at a general ward where those that couldn’t pay their bills will be. Actually, it is not a very comfortable place, so at a point, they start seeing mothers coming with moneys that they can pay half.

I: Oh OK.

R: So, they have a strategy (with which) they take care of them, but because we don’t actually deal with them fully, I mean, as money-wise…Uh huh…..so I wouldn’t be able to say much on that sector.

I: OK. OK, so that is what you know. And then, in this unit or even in the hospital as a whole, what are some of the reporting systems that are available to encourage women who come here to deliver to report abuse? And the abuse, it can be verbal abuse, it can be, you know, any physical abuse…so what reporting systems are in place to encourage these women to report such abuse?

R: Reporting systems….well, most of the time, if they have any complaints about the service being…if they are uncomfortable, mostly, they approach the in-charge on their ward.

I: OK.

R: Or at times, they – some - just go straight to the CNO in charge of the directorate. So mostly, those are the areas they go just to report.

I: OK. And uh, have you received any such reports from these women or even from the directors?

R: To the best of our knowledge, currently, not really. Not really. None at the moment. None at the moment, but I think, at a point, a client became uncomfortable on the ward and her experience was at night simply because we don’t have curtains….

I: Yeah.

R: …..so much cold…so, mosquitoes too – there is (an) influx of mosquitoes into the ward.

I: Mmmmmm…..

R: So they become so uncomfortable and they tend to report to the CNO (laughing) what happens at night. Uh huh…..but in-charge mostly is always around during the day.

I: Mmmmmm…..

R: So, there is another in-charge for the night though but at the moment, they tend….they report to the in-charge and tell us what happened at night. We’ve even seen a situation whereby those with critical cases, they tend to tell us they want to go home because the place is not comfortable because of the mosquitoes and so much cold. But we still counsel them to stay. After all, it is for a while and they get better and go home.

I: Mmmmmm….so you were talking about the fact that if… some patients can approach the in-charges and also the CNO. So, these reporting systems that are in place, do you think they are friendly to all the women who come here? For instance, it doesn’t, you know, it doesn’t ….like the reporting system, can they easily approach you the in-charge, or the CNO and maybe….like the reporting system is friendly to all classes of women, whether the patient is educated, whether the patient is uneducated and whether the patient has money, whether the patient does not have even money to pay the hospital bills and all that? So these reporting systems that are in place, are they friendly to all the women who come to this unit?

R: Yes, they are. Especially the in-charges on this ward, we are not status-biased. So, we embrace them (smiling), listen to them. Uh huh…because it is by listening to them that we see where there are lapses to be corrected. So, especially when they report to the in-charges or the CNO, it becomes a big challenge on our side to quickly rectify such a situation. Because, that client is in dire need, that’s why she decided to report to the appropriate quarters.

I: Mmmmm…..OK. And then, in addition to these reporting systems, what other strategies are in place that help investigate abuse of patients even in the absence of women approaching you the in-charge and then telling you that they have been maltreated or they are not satisfied with this service or that service? What systems are in place to monitor such abuse?

R: Actually, as in-charge of the ward, every morning when we report, we do bed-to-bed handing over and bed-to-bed interactions with the clients. So we ask them what happens overnight. ‘How far have you gone? Are you enjoying our services? Is there any problem? Do you want to tell us anything?’

And even during our ward rounds too…Yes. With the doctors, we also ask them ‘Madam, do you have anything to say? Because we are here for you, we are at your bedside to care for you’.

I: Mmmmmm…..

R: So during these stages, definitely, the patient, if she has a challenge, she will definitely voice it out something and tell us.

I: Mmmmmm….OK. In-charge, thank you very much for having this interview with us. Do you have anything that relates to respectful maternal care that you would want to add?

R: Yes. I think we….respectful maternal care is very, very important because….for instance, we are ready to work, but at times we do not get the facilities or the items to work with. So we need help. We need help. We need help! We need help, especially this – our ward – we really need help.

I: Mmmmm….

R: If we can get an individual or a group to adopt the ward and transform some essential needs, we would be very grateful. We are ready to work: that is why we are here. So we need help. Yes, because at times, requesting it from the hospital takes time and they don’t answer us sometimes. And meanwhile, they know this….. on the nurse or the midwife to carry out everything….

I: Mmmmmmm….

R: So if you don’t do this too, it means you are irresponsible and you don’t know what you are supposed to…what you are out there for. So, the load is so much, and we really do need help. So, respectful mother care, it should continue from generation to generation because others (laughing), the young ladies are coming up, and they will become mothers.

I: Mmmmm….

R: Uh huh…so we should continue to lecture new professionals that come up so that at the very early stage, not when they have passed through to becoming seniors at work before…uh huh…..at the very early stage, we put it up to them. Then from there, use them up before they grow in the profession. So I think respectful mother and maternal care is very good. So we catch them very young – we catch the professionals very young – with respectful maternal care, so they can carry it up.

I: OK. Thank you In-Charge for having this interview with us this afternoon.

R: You’re welcome.

I: Thank you.

SALLY IC 30 MINUTES 1 SECOND.

I: Thank you very much for your time in-charge. Please how old are you?

R: Thirty-seven (37) years.

I: Please your educational qualification?

R: I hold a bachelor in midwifery.

I: Mm tertiary. Please how many years have you practiced midwifery?

R: Twelve (12) years.

I: How many children do you have please?

R: Three (3).

I: Please are you married?

R: Yes please.

I: In-charge please how do you understand respectful maternal care?

R: To respectfully and obediently cater for a pregnant woman without her holding no

grudges against you.

I: What do you do to promote respectful maternal care at this ward?

R: First, I have to supervise the work. Being a rotational nurse, student midwives or mine

own staff (junior midwives and my subordinates) I will make sure they do the right as

expected as respectful maternal care.

I: What strategies do you use to help patients in labor whose actions directly or indirectly

may put their baby and themselves at risk? Maybe a patient will be in a labor whatever

you tell her to do she refuse and you’re scared she’s putting the baby at risk what do you

do to help?

R: The only thing we do is to encourage the patient and also explain the procedure to her

we know labor is painful so we do our best for her to deliver safely.

I: Since you became a midwife here have you had any complain from a patient that a staff

talked to her harshly, pinched or slapped her?

R: Erm…on few occasions, there are complains. Some complain that some midwives talk

harshly to them so as an in-charge I make sure to talk to the staff every morning before

they begin their work in order to put them on their toes.

I: Do they tell you the reason why the midwife talked harshly to them?

R: Sometimes when the patient complains I call the midwife in question to ask about her

side of the story so that we know how to handle the situation. The information I gather

determines how I handles the whole situation.

I: After that do you see any improvement in the attitude towards the patients?

R: When it comes to attitude some will change others will not but when it goes with

supervision you see changes. As an in-charge you can’t sit idle but when you involve

yourself there are certain things they will not do and it won’t happen and also you

correct them when they do something wrong.

I: Have you had any report from a patient that a midwife examined her without seeking for

her consent or permission?

R: Oh erm…when we take vaginal examination (VE) for instance (laughs) the women

themselves will call you to come and check. It is not common for a midwife to attend to

someone without her consent though at times it comes unaware but is hardly to see a

midwife performing a task without a patient consent.

I: Okay. In-charge please how many privacy screens do you have?

R: Erm at the first stage we have two (2) screens, and for scan we have two (2) and other

two (2) screens for examination because each cubicle has two (2) patients.

I: Are the screens adequate?

R: Oh, for now it is adequate because we use it when there’s an examination and we don’t

examine all at the same time.

I: How do you deal with it when you’re in need of shortage equipment maybe screens or

curtains?

R: When there’s a problem we make a request to the business manager and they will supply

us with one.

I: Does it takes long?

R: Sometimes it does at times they bring us screens that are not in used.

I: Okay. So, if you’re in need of screens who do you firstly write to?

R: I first inform the nurse manager then to the business manager and finally to the HOD to

make the preparations.

I: What are the challenges you face when you request for equipment that will make your

work easy?

R: It is very complicated because of that bureaucracy and centralization. You may even

need a kettle but before you’re done with all the processes it can take two (2) weeks or a

month to get it. You’ll go they will tell you HOD is not around, business manager is at a

meeting all those stories and finally if they approve for you to collect money from

accounts office that too not easy. The stress to get the needed equipment is too much.

I: Normally what are your responses to these challenges?

R: Sometimes we improvise to make sure the work is ongoing till they provide what we

need.

I: In-charge do you organize in-service training for new staffs and existing staffs?

R: Erm… for new staffs.

I: Workshop on patient’s rights?

R: Yes, we do it for the new staffs but currently our in-service training for continue staffs

has reduced but we do. It is not as often as first but now when you hear about any

workshop and you’ve the resources you can attend.

I: At this ward do you organize some for the old staffs?

R: Yes, we do orient them even if they’re from different ward we do so they will know how

work is done here.

I: Those that are been oriented what are some of their responses to the orientation?

R: Oh, is not so bad because we put both the old and new staffs on duty so they can learn

from them. Apart from the general orientation, when they work with old staffs they get

to know and familiarize themselves with the ward.

I: Do you also organize in-service training for the nurses on how to treat patients with

respect and dignity?

R: Erm…these workshops are normally organized especially when there’s mortality.

During mortality meetings, if staffs attitude becomes a factor in the rise of mortality we

address.

I: What challenges do you face in training your old and new staffs?

R: Erm the challenge is allowing all midwives at the ward to attend a workshop. If they are

ten (10) I can’t allow them all to attend. If the workshop can be in batches so that at least

the midwives can also be in batches to attend the workshop if not the few ones that will

attend has to relay the information to those who missed it. The challenge is when it is a

one (1) day workshop and know one gets the opportunity to attend.

I: In-charge what do you do when it is reported that patient can not pay for services or any

other concern?

R: Aww.

I: Maybe a patient has been discharged but don’t have money to pay for the services.

R: Sometimes we inform the nurse manager then she will inform the business manager and

maybe to the social welfare to solve it. What I do is to report because as an in-charge

you can’t do things in your own will.

I: In this hospital, who ensures that patient is detained if she can’t pay her bills?

R: Erm to make sure…

I: Yes, whose responsibility is to ensure that a patient who can’t afford her bills is

detained? What unit?

R: We don’t have a unit responsible for that. Report is taken on the number of patient’s

being admitted and discharged so if one misses out they know.

I: When you report to social welfare how do you solve the discharging of the client?

R: It is handled by the social welfare either they settle all or half for the patient to pay the

rest.

I: Taking the hospital as a whole, what reporting systems are available to encourage patient

to report abuse?

R: There’s a suggestion box and some patient do approach the in-charge I think that’s the

only way to report their grievances.

I: Have you had any report from patient that they have been abused by a nurse?

R: It is normally about how the midwife shouted or didn’t speak well to her. For physical

abuse or assault it has never happened here.

I: Do you normally get such complains?

R: Erm…yes but not that much.

I: What do you do when it happens?

R: We call the midwife in question then talk to her when it still continues we have no

option than to query the person or report to the authorities.

I: Do you think the measures putting in place to ensure report from patients are friendly to

all irrespective of you are?

R: As an in-charge every morning you have to move from one cubicle to the other to ask of

patient’s wellbeing and with that they feel free to tell you the complains about how the

midwives treat them. Even among themselves they do communicate about the

midwives so I think that rapport helps them to voice whatever they feel or how they are

been treated.

I: In this unit or hospital, what measures have you putting in place that checks how

midwives treat the patients even if they don’t report to you?

R: I think is supervision when you delegate the work and supervised it helps them to realize

their flaws. The subordinates will help the junior nurses and you as an in-charge will

supervised the subordinates whether they’re doing well or not.

I: What are the things you think will help respectful maternal care that we have not talked

about?

R: Erm as for the workshops though is not effective as first but there are other online

workshops but we need to encourage the staffs to be up and doing is not about only

workshop but we have books that they can read so they can use scientific knowledge to

work successfully.

I: Do you have any advice for other in-charges on how to ensure respectful maternal care

in their unit?

R: Some in-charges do not even question some staffs when they do something wrong and

that does not help because the juniors may think everything seems right if they are not

questioned on the wrong they do. Also, we have to praise staff to encourage others if

they do good we shouldn’t be fault finding in-charges every day.

I: What should the hospital provide to ensure respectful maternal care?

R: They should look at how the bureaucracy system works that delays our request if they

can make it easy to get access to our needs.

I: You’ve worked with midwives is there any attitude about them you will like to

comment?

R: One attitude is that a lot don’t like reading. When a doctor write’s something we don’t

read into details but stuck with what we know already so we should be open minded to

learn new things. Also, the senior colleagues should supervise the juniors to improve the

work.

I: Okay in-charge is there any other submission to add up?

R: Nothing new but I think if respectful maternal care would be implemented for us all to

accept it will help and make work easier for us.

I: In-charge thank you very much for your time and your contributions on respectful

maternal care concerning that of midwives that don’t like to learn new things and the

hospital that needs to make provision on how not to delay request.

R: Thank you too.

VIDA 1C

AUDIO LENGTH (60:47 MINUTES)

I: So Ma, thank you very much for agreeing to have this conversation with me this afternoon about respectful maternal care. And as I explained the rationale to you, I hope you’ve agreed to be a part of this research.

R: Yes.

I: Please, how old are you?

R: Years?

I: Yes.

R: Fifty-four (54).

I: Fifty-four. Then Mama Vida you are very beautiful: if someone sees you, the person might conclude you are in your late thirties. You are cute (both burst into laughter). Please, educational qualification?

R: Tertiary.

I: Yeah?

R: Yes, I do have a BSc in general nursing.

I: Oh nice. Ma please, you’ve practiced midwifery for how long?

R: I will say all my life.

I: Mmmmm?

R: After school, after midwifery, from 96 (1996), I went to surgery, medicine, child health, consulting rooms, theatres and I settled in O&G in 2000 and, I think one (1, effectively 2001).

I: Oh OK.

R: And since then labor ward, theatre, nurseries and others. It’s been a long time.

I: Very long. Which means you started nursing actually in 96?

R: Nursing, I completed nursing in 92 (1992).

I: So it was in 92 that you started practicing?

R: It was in 92 when I started working for a little while…..92, 93, and then I went to midwifery, for a year; one year.

I: Oh OK.

R: Yeah. So it was in 96 that I completed midwifery.

I: Then it means it has been a long while.

R: Yeah.

I: Ma please, how many children do you have?

R: One (1).

I: Please, are you married?

R: Yes.

I: Ma please, when we talk about respectful maternal care, what is your understanding?

R: In this our profession, my belief is the respect with which you will perform your duties as a midwife with your patients, I believe that is what we are discussing. Well, in all you do, you have to make your mind that you will not step on the feet of clients and whatever you want to do for the client, the client will receive some benefits that are helpful to the clients themselves whether it is the cure of a disease, or if the client to deliver a baby, the client wouldn’t experience any negative feelings or thoughts concerning the duties you performed for her. That is how I understand the concept.

I: So let us assume that someone is here, a pregnant woman has come here in labor, what would you (the entire staff) do for her that once she has delivered and gone, you can confidently say that ‘for this woman that came here we were able to offer that person respectful maternal care’?

R: What is at stake is that our job is a little difficult. But when they do come, here, what we first do when we realize you cannot sit down is to greet you, mention our names, whenever you are going to conduct any procedure on a client, ‘I am called Asomasi (so-so and so)’ and then you go ahead and explain what you are about to do to the client for her to understand. This place is not similar to the other side, the other hospitals….

I: Uh huh…..

R: If you approach a client to give her care and she feels she is not ready, if she tells you ‘I am not ready’, here, if you are not ready, we wait, as long as it is not an issue where delaying can prove detrimental to you, then we will wait (if delaying will be detrimental to you, then we won’t wait) but if we wait on the procedure and it cannot be detrimental to you, then we will wait. When you are ready, then you come and we do the procedure. Also, there is no shouting (at clients), no beating (of clients), she calling you for help and you refusing to attend to the call does not exist here. Here, we have bells – door bells – so the moment you ring them, then a staff will appear and help you with whatever you need. So anything you may require, we respond accordingly (perfectly) here so that when the client is leaving, she leaves with happiness after she has gotten her baby.

I: Mmmmmm…..

R: Aha….I won’t bother with you, and all these little squabbles and disputes, I’ve not seen any since I got here. Thus, whatever they came to learn, they are putting it into practice: it is there already and the workshop they came to has become something like a reminder.

I: Yeah.

R: Uh huh. It will remind us that for our work, this is the way to go about it: giving the needed respect to the clients. It shouldn’t matter whether she (the client) is rich or not, here, we have to do the right thing for everyone in the proper way so that everyone can be satisfied. In addition, food..the issue of food for clients should also be treated in the proper way so everything is rightly and properly done. We serve the food ourselves, and we take it to them. And we inform them that ‘please, food is here’. In case a client doesn’t like the food served, we will not stand around and complain ‘you are too pretentious’ or anything of the sort or hurl any insults, but the next batch of food that may be served, you can tell the client ‘we didn’t know you did not like the previous meal, so next time, we will do what is necessary for you’, and we go with that. So I feel that is the right thing to do. And we work together. I tried doing cubicle nursing, but I found out that, looking at my staff, if I go that route, the numbers will not be enough. Uh huh…so we work together. The assistants and all the other staff, everyone do their best and we work together. As a result, since I’ve been here, I have never observed a nurse and a patient in a quarrel.

I: OK.

R: Aha….I have never observed such an incident almost for a year – I will be here a year in July. I have never seen anything like that.

I: OK.

R: So, those who are specifically brought here are (the staff) those who are down-to-earth, those who are respectful….

I: You are talking about the midwives?

R: Uh huh….that is how I have to put it…those who are respectful and give the needed deference to clients. This is because it is very important people who show up here…important personalities, wives of ministers, the wives of chiefs and their daughters, nurses and doctors, these are the people who come here. So when it comes to the respect, it is all good when we go that route. Even the blessings of God that can be bestowed, they are a lot.

I: Yeah.

R: So, promptly, we answer their questions and answer their calls.

I: OK. Well, as an in-charge, you said you’ve been here for about a year, and since you’ve been here, what are some of the issues that maybe you’ve tried to put in the right to make everything work properly?

R: Well, since I have been here, we use gadgets to perform our duties and the monitor is only one.

I: Monitor for?

R: Monitor for patients…..we do the recording ourselves.

I: Oh OK.

R: Uh huh…when they are done with the surgery, we do that ourselves. So when there are two (2) or three (3) surgeries, we are stuck. As a result, I requested for more of the monitors and a CTG machine that we use to check the FS.

I: Mmmmm….

R: There are some clients who want it to be continuous, and also for you to print out the results, and since we do not want one individual to exclusively have access to the machine (CTG), I have also requested for more. And I also observed the curtains to be old….

I: Mmmmmmm…..

R: The curtains were very old. So I had to struggle with them for about seven (7) months, and then I got them. So I make sure, every day, once I report in and sign my name, and I looked through the accumulated reports and enquire from the night nurses if there is nothing urgent, I then make sure everything we need to work with is available. God being my witness, when it comes to this place, because of the amount of money charged, once you request for equipment they will give it to you but it will take a long time.

I: OK.

R: You will eventually get it but you will be frustrated a little bit. If you don’t….the follow, if you go there one or two times and you stop, they will also relax. If you persist and you push, they will also give it to you also need to work with.

I: Oh OK.

R: Concerning stores, unless there is a shortage at the stores and then that can affect us. But I have also planned that I will ask for more than I need, so that when the shortages occur, I will fall on those ones and use it to work. We’ve gotten some of the gadgets. It was the PPE’s that was a problem, especially with masks once the COVID hit but nowadays, since it was reduced to eight (8) Cedis, the girls have even bought some themselves.

I: What girls?

R: The midwives.

I: Oh OK.

R: They have bought some and when they come to work, it will in their bags. Every week, we are given one box like this one (indicating), and when we share it, each individual gets three (3), and that is not enough. So I have advised them that they should get extra ones by themselves for all of us to protect ourselves. Thus, concerning the gadgets we need to accomplish our duties, it is OK but here, the billing – the process through which the client will get the bill and pay – on weekends, I have a problem here. That is my main problem here.

I: Mmmmm….

R: That and our mothers who go from here to the MBU to visit their babies every two (2) hours: it is very deadly in the night. So we were trying to even get the pediatricians to come over to see the babies. In that regard, I would have rather found the space here and provide two or three (2 or 3) cots to use for the babies….

I: OK.

R: ….so that from time to time, they (pediatricians?) will come and do the reviews. This is because most of the midwives here have gone to the MBU, some have even gotten experience for three or four years, so the experience is there. Which means that I can use them for the two issues (???), but when I made the request, it hasn’t come to pass. It never materialized because the pediatricians are not ready to come (to the ward). For some of them, to come and observe (the babies)…..it seems they started a program like that (in the past)…..

I: Oh OK.

R: …but on a closer look, you will find out the cost of treating the baby is even much more expensive of the mother being hospitalized here. So they just went back to the old system. So they have to go and then see their babies. Sometimes they will go, and the moment they get back here, they will collapse.

I: Oh!

R: So that is our main problem. Apart from that, I really don’t have any issues. Please….thank you….that kind of talking and such….’Oh, I will make sure things are taken care of’ but ‘Oh, why are you bothering me?’ and stuff like that…

I: Oh, those (kinds of behavior) are not permitted here. Mama Vida, you were saying you recently requested for screens….

R: Curtains.

I: Curtains! In your opinion, are they adequate?

R: Yes. I even have a surplus for, I think, even four (4) in a room.

I: Oh OK.

R: I didn’t even use that of the labor ward, so those are also available.

I: Oh OK.

R: When these are dirty, I can use the reserves for, you know. If I use one of the four-in-a-room for a room, then I can take it (the dirty curtains) down and replace them with it. I have the old set there, so I can also it for them….

I: So it means when it comes to your place….

R: I have two sets of curtains.

I: Two sets of curtains, OK. Which means in your place, you have enough screens for privacy.

R: Yeah. We don’t…..we use the curtains for screening, so I have them.

I: OK. Well, Ma please, let’s say a mother is here to have a delivery. You’ve observed that either she herself is in danger or the baby might be, but the patient is not cooperative. In such an instant, what will be your approach or what do you demand your nurses to do?

R: In such instances, we do an explanation of what is going on; if you do not educate the patient on what is going on and what you want to do, she will not understand (and cooperate). Sometimes the CTG, if the patient gets here and it is a fetal distress, we put her on the machine and increase the volume. She can hear the sound. She can understand when it gets to a point, the volume goes down. So the explanation of what is going on to the patient will lead to her calming down. Uh huh…but if she does not know what is going on and she feels when you get here, you are supposed to be given an infusion (for instance), fetal distress, we give infusions, we give oxygen, and then we call the surgeons to report (the conditions going on). Thus, if you do not explain, she will not understand. But if you do explain and if you have a vivid way to show the conditions to her, you show it to the person. Therefore, you explain that this is what is going on: ‘if you do this, continue to do this, this is what is going to happen’. So, in such instances, you have to explain. So, I’ve counseled them (the staff) to exercise patience to explain whatever procedures they may want to do and what is going on to the patients, and then the patients will agree with you.

I: And you were talking about the fact that since you’ve been here, you’ve not actually seen anyone mistreat a patient, or if you ask a patient to push, she is refusing to push and as a result the midwife may spank the patient a little or some staff may even go and hold the nose of the patient to demonstrate that (laughing) ‘can you understand how the breath of the baby is being withheld….’ (interviewee joining in the laughter)….Ahaaaa…or the patient being bound on the feet so you can get access to….

R: No. For me, even the stirrups here, I do not use the straps.

I: You don’t use the straps, OK.

R: I don’t even use the straps to restrain the (15:14). And if she (the patient) cannot push, that was what I was talking about: if you do not educate her about what will be happening, she will not understand. Sometimes, I do side with them (the patients?) because she becomes exhausted. Exhaustion occurs, and then you explain other options you can use to help her; either you give her Synto or you’ll perform a vaginal (vacuum?) for her. If the person can’t push as…but if she can push….I think, last two (2) weeks or so (the previous two weeks ago), the client’s husband was around…

I: OK.

R: Instinctively, something told me to inform the husband to come and stand beside her, and talk to her. I’ve done that twice, and it worked.

I: Mmmmmm…..

R: ‘Talk to her that the baby’s breath is being withheld, and she is refusing to listen to us when we explain the situation to her’. The man was with us here for a long time, we allow them to stay here, at the second stage room.

I: OK.

R: After the delivery, one of them even nearly collapsed (both burst into laughter) (16:24-:26) and all that.

I: Ah…it is not easy.

R: The woman was not pushing, thus the strategy I used for the husband to come in and he talk to her and then massaged her and all that. So, while the husband did his part, we were also encouraging her.

I: Mmmmmm….

R: Here, we do not beat anyone. Eii! (exclamation for surprise) If you do anything like that, it will get to the top (top management) immediately. They just have to climb (the stairs). You see? So we don’t do such stuff here. So we encourage them and let them see the stage in which the baby is…

I: Uh huh….

R: Ahaaa…to encourage them to understand that if they delay too long, all the complications that can ensue, we talk about all of them. Uh huh…then you realize she will use all her strength to push and we will use something, Synto or something else, to help her so she can push.

I: Well, before you came here, had you worked in the labor ward?

R: Yes. Yes.

I: And in that place, were there instances of, for example, people being beaten or other things that people might do… did you ever hear about such things?

R: Labor ward…personally, I didn’t hurt the women but I was a midwife.

I: OK.

R: But others were doing it. Me, naturally, such things (considerate behavior), it is there already; I’ll never do such things, but others were doing it. To slap the thigh of the patient (demonstrating) or ‘Madam, I will go and sit down and leave you here alone’ and you will have to push by yourself….especially the night duties…

I: OK.

R: Maybe by the time they get to the ward, they are already tired and sleepy, all that. And if you are standing by the client and she will not cooperate, ‘Sister, if you do not push, I will leave you and go’. Those days, some clients had to deliver the babies on the floor and such.

I: When some patients had to be put on the floor?

R: Yeah!

I: Well, I even when I came here (when I started working here?)

R: The corridors leading from the theatre all the way down to the labor ward, there were mothers lying on the floor.

I: Yes, there were some mothers all over the floor. That I even came to meet those conditions.

R: You delivered on the floor, and got this and that process done….and sometimes it is frustration; you the midwife is frustrated, which one are you attending to? So we will deliver all of them (on the floor) and we will later come back behind and be removing the placenta and other get other things done and then clean them up. So when the workload is also too much….there are staff who cannot handle stress….

I: Mmmmmm….

R: …so that is the she resorts to, to abuse the patient or she may insult her verbally, or she (the nurse/midwife) may hit her (the patient) or she may get into a quarrel with a relative. For such incidences, a number of staff did them. But these days, since they moved down here (indicating), if the place is full, it is full….

I: At the BECA ward?

R: Yes.

I: OK.

R: You are referred to somewhere else if it is not an issue that requires a specialist attention. We will transfer you to, say, Suntreso or somewhere else for you to have the delivery.

I: OK.

R: So that the space….you don’t go there and see clients on sitting on chairs over here. The beds are limited and all that. Thus, it has gone down (the bad incidences have gone down), being beaten and such things, it has gone down. But previously, it was there.

I: Mmmmmm…..

R: Yeah, it was there. For the period between the past three or two years and I think the workshop too has helped. It has helped indeed. It has helped. It has helped a lot.

I: Mmmm…..and Ma, since you’ve been have, maybe you’ve gotten a complaint from a patient that maybe a nurse came to perform a procedure, and when she came, she never said anything to the patient and just went ahead to perform the procedure?

R: No, no, no…that is what I have already talked about: I have never gotten a complaint in that regard since I’ve been here. Especially, there is the possibility that this place can get very busy and the attention that should be given to a particular patient may have been reduced, and the patient might talk about it ‘I have not been given my medication’ or ‘since I came back from MBN, I’ve not been given my food’. These are the two (2) main issues that they talk about here, but nobody has ever made a complaint in the line of ‘I’ve been insulted’ or ‘she didn’t give me the expected respectful treatment that I should be given’, maybe the nurse just arrived and wouldn’t even say ‘Please Madam, I am here to give you an injection’ but just ‘turn around’….

I: ‘Give me your butt’……Mmmmmm….

R: When you get to a patient, you have to introduce yourself…’my name is ‘this’, and the time for your medication is due, you’ve got to take this and I am here to administer them. Please, are you ready?’ If you are not ready, she will get back to me and report ‘Ma, she says she is not ready yet’. A doctor once came here from Suntreso….

I: OK.

R: The moment you get to the bedside, you would be told ‘I am not ready’. I was going to pick up her baby to give it a bath, the lady said she is not ready. The second time, her medication – it was an IV and flagil – she said she wasn’t ready and I told them that ‘If you get there and she says she is not ready, don’t be worried about it’.

I: OK.

R: ‘Come and continue’. So the second time she informed us she was ready, the staff was also busy here, and so I told the staff that had to attend to her to inform her that we were also busy here, and the delay is not a punishment or anything of the sort because she didn’t allow them the first time.

I: Yeah.

R: Yeah. Such things are not allowed here.

I: Mmmmmm….eh, concerning the curtains, I know we’ve talked about them but when you were in need of the curtains, any other challenges that you faced?

R: Concerning those curtains?

I: The curtains, because you need those to ensure privacy?

R: Yes.

I: Ahaaaa….

R: When it comes to the curtains, truth be told, I had a hard time. I really had to struggle for those, to the point where I had to physically go to the HOD, the then HOD, I went to his place….Dr Asubonteng.

I: OK.

R: I went to see him and had a conversation with him on it…how urgent it is but at the time, the information given was that the directorate didn’t have the funds so we have to wait for a while. So what I did was, just as I told you, I would take one room’s…..

I: One room’s curtains?

R: ….and I will use it to the laundry and wash it.

I: OK.

R: I will get it back same day and then I will hang them back.

I: OK.

R: I did it like that for a while and then when the COVID hit, the attendance was very low.

I: Aha!

R: The attendance was very low, and as a result, that was the time I got the chance to take down three (3) rooms….

I: Ah….at a time…..

R: At the same time, together, and I took them to be worked on. And I saw that when I do it like this, it is OK but if I had gotten cases, I would have been in trouble.

I: Mmmmmm…..

R: You follow?

I: Yes.

R: Thus, for you to take such a decision, the place has to be less busy for some time before you can do such things.

I: Mmmmmm…..

R: My problem was that it took a long time before they came to measure the length and all the dimensions….

I: And that was the issue you said took about seven months?

R: Yeah. …. To the contractor…they contracted someone who sells curtains, and then the contractor also did the orders for us and then I took down the old ones to get them laundered and I’ve saved them as a backup with the view that when these get dirty, we can use those ones. Thus, it just took long, and the problem was that they didn’t have money.

I: OK.

R: At that time…they didn’t have money, and sometimes, their hands are tied, you can’t force through. But if you need things urgently, you will have to lend them money, and the loan, (chuckling) it will take a long time for you to be repaid. So that is what happened.

I: Mmmmm….

R: It took a long time. They gave me the assurance that they will provide them, but not now.

I: Oh OK.

R: So it was after about seven months that they brought the information that the curtains have been brought in.

I: Ma please, in this place, if you get new nurses or midwives, and even the midwives who have been here for a while, training, do you have any training as a ward or even as an in-service training that educate the midwives about the rights of the women who come here? Let’s say the clients who come here have the right to…..

R: The rights of patients….well, since I’ve been here, I’ve not done anything in that line for them. I do not know about my predecessors, but if you are new here, I put you under the tutelage of someone I know to be very competent and the procedures and other things that go on here, you would be taken through them as an orientation. She will take you through them….this is what goes on, this is how you should comport yourself. Well, I intentionally partner you with someone so you can be taken through all the protocols. If you have any problem, I will tell you ‘report back to me’ so we can know what to do about it.

I: Mmmmmm…

R: But personally intentionally organizing something here, not yet. I’ve not done that yet.

I: OK. But what about in-service training? Have you had anything of the sort?

R: In-service training, yeah, I’ve been to a lot of them. Unfortunately, it was the one organized by your people that I wasn’t able to attend because this place was very tight. Some of my people were sick; a few contracted COVID.

I: Mmmmm……

R: A few got COVID, others were pregnant, others were going to school and such. As a result, I was personally standing in here and that is the reason why I could not attend your workshop. Chief also told me you will roll it for some time, so I was waiting for my turn…

I: For some time…but it never came to pass.

R: So, when it comes to the personal ones, I have not done one but that is the strategy I use here: I put you under someone when you get here to teach you what you are supposed to do. For the rest, our ethics, patients rights and the rest….such workshops, we’ve ran several of them.

I: Mmmmm….Yeah.

R: (27:05-:10) I told her that, those things, teach her, for they use it a lot here.

I: Yeah.

R: For a lot of the ‘I know my right’ people do come here….

I: Well, this is the Special….

R:….if you are not careful, you will just plunge the hospital into a suit…problem, you know, they will take you out there in a minute. They will take you out there. So that is just how it is.

I: OK. But in your opinion, the training the nurses go to and those who are mentored by the experienced nurses to be coached and mentored, once they get the training, what is your take on it? Do you see them put it into practice?

R: They really put it into practice, indeed. Grace, there is a young lady here, ….she is not even exactly here, but rather in the dark room, I came to meet her there (28:00-:05)….

I: Oh OK.

R: ..and she has built herself up over time to win the best nurse (award?) in this ward.

I: Right here?

R: Yes.

I: The one whose picture…..the wearing a blue (dress)?

R: Yes. This same mentoring you are talking about. Thus, I have not yet encountered any problem with such issues. There are people have never been to such a workshop like yours, and if you get here and we realize you’ve never been to such a workshop, we will tell you the little that we do remember. And I used to have the patients charter here….

I: Mmmmm….

R: Yeah. I made my mind to run copies for them. It has been misplaced among my things; I can’t locate it. If we do not look at things from that perspective, we might end up doing things in a very wrong way here. Therefore, it is important for us to touch on these things: patients rights and the code of ethics and all that.

I: OK. But please, in regard to the training given to the new midwives, you getting them coached, is there any challenge that you face?

R: Well, the problem that can occur is that, along the line, something might happen, for instance, she might go on leave, and you have to look for somebody else to do it, or I do it myself.

I: Oh OK.

R: And that is the main problem here, apart from that, I do not see any other problem related to this. We see that, the individual, even if the person is averse to working (effectively) from wherever she is coming from, once she gets here and realize or observe how things are done here……..and I believe in motivation.

I: OK.

R: I’ve even got some right here (indicating….motivational quotes??). I believe in it. And there are instances…… it is the patients who have been admitted here who bring such things back to us (as gifts). I make sure that – they will tell you – if there is anything to be shared here, I share it equitably. I do not make distinction that this person or that person is an SMO, there are people who will use the rank distinction to distribute such things. If someone gives us a gift of money and I have to divide it among the staff, it is for all of us; whether you are an SMO or JMO, everyone gets an equal share. This motivates them to learn new things.

I: Mmmmmmmm….

R: So I’ve not yet gotten any challenge in this regard apart from the fact that the person being a mentor can be absent due to certain issues like changes to the person’s schedule or going on a leave or, a leave is a leave, whether it is maternity or sick leave or whatever. Ahaaa….all such things, and if they have any challenge, they fall back to me…..

I: OK.

R: …and whatever I have to do about it, I do it.

I: What about the training they attend, for example the in-service training we organize, you might realize all the midwives would want to attend. How about that? Is there any challenge?

R: It is a challenge, but then, I take cognizance of those who have been there before. You realize some of the workshops are a repetitive kind of workshop….

I: Yeah…

R: ….Aha, so when I am aware you’ve been to such a workshop already, I will not allow you to go back. Those who have not get gotten the chance to be there, I will push them to go for the knowledge. At least when she gets back and it will give you a reminder, just to remind you of certain things that were taught there….

I: OK.

R: ….it would be more helpful than allowing repeat attendances while somebody is ignorant about the whole thing, aha. So that challenge is there, but I try to sort – I pick and choose those who really need the training and make them attend. And I make sure those I am denying understand (the reason they are being denied this time) too, with an explanation. Everything that goes with an explanation makes the people relaxed. Therefore, that is how I go on with our team

I: OK. Ma, I know that even though this place is a special ward, have you ever had a report since you got here that a resident, who is supposed to be discharged has not been able to because the amount of money on her is not enough or she doesn’t even have money to pay for the services?

R: Well, before you come here, we make sure everything is clear to you. Right now that we are having this discussion, there is a resident, and I will intentionally go over to her and ask her, because it will take her some time before the procedure she is here for is due. When they get here, we inform them ‘this is the cost of the procedures, if you are her for this procedure, it is three (3) days, if for that, it is one (1) day, if for this other one,…..

I: So how much do you charge approximately?

R: ….and the bill is this much. Here, how do I even put this? It is an already prepared thing; it is a package.

I: Oh OK. OK.

R: And each package has a bill attached or given. So when you come here, we tell you ‘this is the bill’ and if you cannot afford it, we will allow you to go to the place that you will be comfortable (with the bill). There was an instance, one instance which I remember where the amount of the client was not up to the charge. It wasn’t enough, just as you said. Because it wasn’t enough, what we did was that she would gather herself together…..what was left (of the amount to be paid) was about four hundred (400 GHC); she had already paid almost eighty percent (80%) of the bill.

I: OK.

R: So she will gather herself….we were lucky to have that problem in the morning, so by the evening when I was about to leave the ward, they brought the remainder of the bill to add up. And there was another one with a hysterectomy: we nearly lost that lady. And they did a herniorrhaphy in addition with the hysterectomy. I didn’t know it was the responsibility of a certain organization to come in and foot the bill for her.

I: Oh OK.

R: Thus, when it was time to discharge her, she didn’t want to go. ‘Please, let it be tomorrow’. Let it be tomorrow…about three or four times, and then I said ‘No, there might be a problem’. So I went over to her and started a discussion, ‘Sister, where I live, there are a lot of one-man churches surrounding me that prevent me from sleeping, so since I’ve been here….

I: Mmmmm….

R: And I replied ‘But that is not a good solution (reason) for you to stay here. You have to move. If they are disturbing you, look for another (different) place and move’, not knowing it was CCC (Christian Charismatic Center?) that were sponsoring her.

I: Oh OK.

R: So any amount that you present, they will pay.

I: Oh OK.

R: So in her case, it delayed a little bit. And one doctor who was in charge of the financial issues came and told us that they (CCC) will come and pay the bill. And I said….

I: From her (clint’s) church?

R: Yes. She is here. She is the head of department of one of the units.

I: Oh OK.

R: When she informed us, I said ‘No’, and she said ‘It is even in a cheque form’, and here (Special Ward), we will not accept a cheque: they will have to go to the main revenue department to get it worked out. So, she is giving me his word - I should let the lady leave.

I: Oh OK.

R: I should let the lady leave but she will bring the money. That is one instance that I’ve been involved in….

I: Since you got here.

R: Well, true to her word, they brought the receipt to me just the following morning, but it wasn’t the patient’s name that was on the receipt; they did it manually because the pastors wanted to know that they have really made that payment in the name of CCC.

I: Oh OK.

R: And it seems it was a bit of a problem taking the patient’s name off the (unpaid) bill but when I went to see the accountants, they worked it out for us. So when it comes to the billing issues, in this ward, we don’t get issues where a client may not be able to completely settle a bill and we have to bring in the welfare (department). I don’t have those kinds of issues here.

I: Oh OK.

R: But before they come (clients), their doctors instruct them to come here, so we can take them through everything. I am trying to come out with even the bill and other things that we are going to implement. I have made some few changes to the list, and I have supplied the bills on the back of the list.

I: Oh OK.

R: Aha…I am trying to upgrade it, update it, so that other things can be added to it. You see that the number of days for each (day?) is not added? (Indicating), so I will have to redo this.

I: Oh OK.

R: So once you take the list, then everything….the information on there. One instance, just the one discussed….

I: That is the one you’ve encountered. OK. But aside your unit, the labor ward or the darkroom….that is where the delivery is done, right?

R: Yes.

I: Have you heard anything of the sort there? Maybe, there are clients who cannot afford their bills, and they are detained?

R: I came here from the darkroom….

I: Oh OK.

R: …(38:18-:23) One problem that worsens it is that when their babies are admitted to the MBU, they are more concerned with their babies, so the little they can gather, they will rather spend it towards discharging their babies, then they will come and sleep at the ward. You will try and try and try (to get the client to pay for her bill in order to be discharged) and if you are not lucky to get some volunteers who come around to foot the bills of patients, and you transfer the patient to the social welfare department, they will dilly dally with it for a long time. There was one service person, there was this girl who was here, a national service person, once I call her, she will run and tells me ‘Mama, I am taking the issue to the HOD’.

I: Oh OK.

R: She will stay on top of the situation until it is resolved, and she will bring it back so we can release the patient, so the patient can go home. Later on, the patient will gradually pay the bill off, little by little (amortize). Once they pay it off, they would be told to come and show me the receipt. So their names are on the list. We have to, you know, clear them. And we use it (receipts) to clear them from the list. So, it is happening on the other side, it is really common over there. But right here, the term is ‘If you do not have the money, you do not come here’.

I: Yeah? Of course. When it comes to the Special Ward, yes.

R: If you do not have the money….and it is your own choice, you decide to come here. It is not as if you are forced to come here like on the other side where you do not have a say as to where you want to be admitted to. So here, I do have that problem but on the other side, it is there. Ah, there are some people who will stay in there (admitted) for a long time, and by the time you come back from one weekend, she would have ran off!

I: She would have run away home.

R: She would have taken the bill with her.

I: So in such an instance, what do you do?

R: Our social welfare department will trace them.

I: Oh OK, they are able to trace them.

R: There are some that you will never be able to locate. You will never find their whereabouts. But there are some they can trace, and they will retrieve the monies and bring them back. There are those who claim ‘I am going home to get the money for the bill’ but they will not tell the nurses on duty. The moment she wakes up and realizes everybody is gone, she will vanish.

I: Who? The mothers?

R: She will go, and then return, because her baby is still here.

I: Oh OK.

R: There are even those whose babies are not here, but surprisingly, you will later see them come back here with their husbands to come and pay off their bills; those with a correct (clear) conscience (both laughing) but those who do not have a proper conscience, they can even leave their belongings here and vanish.

I: And they will go….

R: Yeah, they will leave. It is common on the other side, but here, it was only the case related to the CCC lady.

I: OK. Well, concerning the darkroom when you had to deal with the social welfare – when you had to inform them – what are some of the challenges that you faced? In working with the social welfare department….

R: Social welfare….well, I was talking about them dealing with issues…and sometimes, it is not their fault because it has to go to the HOD’s office. And then he has go through the whole issue before he can approve it. There are times he will not even approve it, and he will return the bill and recommend that they (who?) should take a second look and see if something can be done about the amount on the bill for the patient. Then the patient will pay, and leave. There are those who can pay a portion of the bill but cannot raise the rest, and then there are those who cannot pay a dime. So sometimes, the delay is not from the social welfare people but our bosses who must see to it that the work on the bill is completed so we can release them, they are not there. There are instances where such bosses had travelled, two to three (2-3) days, maybe towards the weekend. Today being a Thursday, the boss is gone to Accra…..

I: Ah…..

R: You have to wait….the patient stays on admission till the following week before you can get him/her to sign off on it and you can then release the patient. And if it is not signed, he – the social service worker – cannot give you the letter to be given to the patient to go home. Without this, people are suspecting that they are taking money (bribes) and such…and because he (the social service worker) may not want to be accused of such behavior, he will see to it that such things do not happen. There are patients who may want to pay a portion of the bill in order to be released, because it is common to have a very large bill – 6000 or 7000 (GHC) – and if the patient cannot raise the amount, it makes it so difficult for the HOD or whoever will work on it to endorse it, you see?

I: Mmmmm….

R: So, at least we will talk to you…we will talk and talk incessantly to you for a long time before sometimes the patient is able to raise a little amount to come and reduce the bill before it is forwarded to the social welfare department to work on it. Thus the signing, and making the part payment can make the whole process delayed. That is the nature of the issue.

I: Mmmmm….and Ma please, in this hospital, or even in this unit, what are the systems you’ve put in place to encourage the patients in the event that maybe a midwife did not treat the patient right, what are the systems in place to enable the patient to feel comfortable to come to you and say maybe ‘this midwife or this staff abused me’. What are the systems are in place?

R: Systems…remember I said when we do the orientation, we add the information that if anything happens and the client is not happy about it, it should be reported to me.

I: Oh OK.

R: Report it to me, and even if I am not here, the leader, the shift in-charge, they should go and report the issue to her. That is the main system in place. And also, I have a box on top of my thing (indicating and chuckling)….

I: In the changing room?

R: No, on top of that desk (indicating) in which they can place their complaints.

I: Oh, in that box….

R: Aha, but it is not labeled as a complaint box.

I: Oh OK.

R: You would be informed that when I am not here, you can place your complaints in there. Since I’ve been on the floor, I’ve not seen any.

I: Oh OK.

R: There are clients who will call you personally that ‘she delayed in giving me my food’ or something of the sort, and then I will talk to the person involved.

I: You will talk to the patient or the midwife?

R: It is the patient who is telling me that her food was delayed….it is possible the fault emanated from the kitchen.

I: Oh OK.

R: And in such a situation, you will have to explain to the patient that the issue stemmed from the kitchen, the kitchen staff did not get here on time. Even this morning, the bread and food (breakfast) brought, there was no egg or anything to add to the bread. And when I went out to enquire about it…it was around nine (9am), and the sister involved, she had gone to the MBU to check on her baby and when she got back, she was looking for her food. She said she’s not been given any food. And when I asked, I was told the night nurses claim their door had a problem with the gate, and they had to wait for the technicians to break the door down in the morning for them to get access to things and all that. So, in a situation like this, what I recommend is that if you have any complaint, confide in me and if we have to discipline the perpetrator…either we write a (45:48querry?) or we give the person a verbal query and all that. The discipline protocols set up here, we use them but I am not so stern on them because if you start treating people that way, if a patient is admitted here, she (the midwife/nurse) will not attend to her, and she might be the only one in duty, you see? So you have to talk to the person nicely, just in the hope that she might understand if she acted wrongly, you see? So she can go and apologize, and the work can go on smoothly.

I: OK. So I can take it that complaints, you were saying that you’ve not gotten any complaints so far….

R: No.

I: And when you were in the darkroom, maybe you got a few complaints from those who came to deliver, can you give me about two (2) examples.

R: Well, when I was in the darkroom, a few came (complaints?). We had a few. One of them was a psychotic patient, and we didn’t know….

I: Oh, OK.

R: It was not (47:03-:05); she was already psychotic and she came to have a delivery and developed a problem. As a result, she was still in admission. Every day when I got on the ward, when she narrates her story, it was as if it didn’t make a lot of sense to me.

I: Mmmmm….

R: So when the husband came in, I was asking him that ‘is this your wife?’ and his response was ‘Sister, hmmmmmm……she was not like this when I married her but she has a mental disorder, so I am pleading with you….’

I: Mmmmm….there is a problem.

R: Well, she fought even with all the cleaners: she would be cleaning up the place and going up and down. You know how they are….

I: Mmmmm….

R: Yeah. A psychotic person will never sit in one spot, but one who is depressed, if you sit her here, you can travel to Accra and back, and she will still be here. She moved up and down, stepping on stuff…well, she did things … and she was constantly complaining. Ah, every day I came to work, she had a new complaint. And I later realized something is amiss. But the other one had a genuine case, in that her belongings….in that place, sometimes we move beds, or we change patients. And her stuff, she claimed she cannot find some of them but the way she said it, it made the midwives a little angry, and they said told her their minds. So by the time I got here, there has been a quarrel in the ward to the point where some doctors are aware and they had to calm nerves down. And when I finally got here, I said ‘it is true, yester, when I was leaving, she was lying down here, and have you really searched the place? It could be that it is the locker that someone has taken….

I: Has changed….yeah.

R: …and has stuffed her belongings somewhere’. And true to my word, when we checked the locker in question, her belongings were in there. Suppose she had hit someone during the scuffle, or if a midwife had maimed her during the exchanges, it could have led to something bad. So it is there, a lot of confrontations.. ..between midwives and midwives, between patients and midwives, relatives…it happens. They happen, but what I have come to understand is that they are minimizing….it is minimizing a lot, but formerly, it was bad. There are instances when they had threatened that when ‘Asomasi’ comes, what did she say? ‘I am going to call my gang’….

I: it was the midwife who was going to call her gang or the patients?

R: The patient relatives….

I: Oh OK.

R: Uh huh….

I: Eii?

R: You have to talk to such a person with that bad motive so that things can come to a close. If I realize things are way above my head, Chief’s place is right around the corner, and I will get in there. ‘Chief, this is the issue at hand. What do we do?’ Maybe he will do this or that, and report back to me. Thus, once in a while, but not daily….but sometimes when the complaints come, we have to properly interrogate the issue and see if the person is completely ‘correct’ (sane) before if we have to take any action, then we take it.

I: OK. Ma, you were saying that when the patients get here, you tell them that if they encounter any problems, they should tell you. There is a system that you have put in place in case you are not treated properly, the patient can report to you. That system you are using, how friendly is it to the women who come here in the sense that it does not matter whether the person is educated, whether the person is sick or someone who is a little less endowed (financially)? Are the systems friendly?

R: Yeah, the system is working, in that, the patients themselves have started giving complimentary comments about the midwives. And the compliments too are a kind of complaint, you know?

I: Mmmmm….

R: Uh huh….if you treat people properly, it is a compliment, it is the good in your actions if you do things right. So far, since I got here, I have not seen that anything has gone wrong or anything is amiss. I see it as friendly as possible for both the midwives and the clients. Uh huh…things are friendly for now. I don’t have any issues. I am looking forward to getting a new ‘something’ to add to this (the already existing protocols?) to make the duties progress much better for all of us. I have to sit down and think about something to add on, because this alone might not help some individuals. You know? There are some people who might feel ‘I should come and report to you? I will not. I am keeping my thoughts to myself, and I am gone’ and that is not the best.

I: Yeah.

R: Uh huh…so if we have to make a suggestion box and place it somewhere and if they can put their complaints in there for us, we would appreciate it. I will think about something. I will think about something, and then, to see what that one will too will bring.

I: Mmmmm….Well, please, when the mothers come here, you tell them that if they have any complaints, they can either report to you or the suggestion box is there…

R: Yeah.

I: But is there any system which is in place that allow to monitor abuse, or if a midwife does not treat a patient properly, what system have you put in place that even if the patient does not come to report to you or if she does not write and put any complaint in the box, it still helps you to monitor that all patients are treated respectfully?

R: Well, I do sometimes go personally to the patients time to time. This morning, by the time I got here, they had already done the handing over. One of my pet peeves is to not get the handing over. Uh huh…I go to them one on one to see if there is a problem. Somebody will find it difficult to come to you.

I: Oh OK.

R: So most of the time I go personally around. I go around and ask ‘is there any problem?’ I have not heard anything yet. It is not like I sit here and wait for them to bring their problems. There are some patients who will never come and tell you, but once she gets out of here into the public, she has a different story to tell. Well, the staff in the whole time, someone can intentionally go like ‘Ah….the O&G In-Charge who is new, that is her?’

I: Mmmmmm…..(smiling)

R: Some intentionally ask others in front of me and if I hear such comments, I will respond with ‘Yes, it is I. It is me right here, standing here’ (both laughing), which means that tales abound out there, whether they are good or bad, I don’t ask about that. So I go to the patients myself and I ask ‘are things progressing nicely? What are the nursing doing for you? How are our meals? Were you able to sleep?’ conversations like that, so that if something is bothering her, she will let you know, maybe ‘Oh Ma, I couldn’t really sleep. The surgery I am about to undergo, I am scared’.

I: Mmmmmm….

R: But when it comes to being disrespected by someone, you yourself, you will not go scot free.

I: Yeah.

R: Uh huh…so whatever I have to do here for the work to progress, they themselves are aware of my position. So they will not do it (bad behavior). They will not even contemplate it. That is another step: I personally go to the patients from time to time and talk to them about how they see us and all that. It is those here that when they do come, they are very difficult!

I: The patients….

R: Doctors and nurses.

I: Ah….

R: Ah……when I get here, I am like ‘please, everybody should calm down. Take things slow….and get your remedy and your healthcare results here, so you can go home and peace will prevail’. When she is gone, it is not attached to you (whatever happened between the staff and the client will not be indelibly marked on the staff). Some people can ‘start’ like that…..she knows that she needs this and that…but she will request for each one at a time, one at a time….the suppository that she can place in there, you have to do it for her.

I: Mmmmm?

R: Her baby is lying in the cot beside her bed, ‘come and pick the baby up for me’…she is doing nothing, right?

I: Mmmm….mmmm….

R: It is not as if she is she is not ambulant, or she is weak. For such a client, you must understand what she is communicating to you and you prepare yourself beforehand. And that is what I tell them (the staff). Well ‘Ma, as for this new patient….’ and my response is ‘you keep your cool’, this work, which is how we execute.

I: Mmmmm….Ma please, concerning this conversation we’ve had about respectful maternal care, do you have anything else you might want to add to it?

R: The little I may add to it is that, whatever we may have failed to do, even though it is our responsibility to do them, my daughters and some of my younger siblings still needs them, the workshops, that you run some more of them….

I: Ah….

R: Uh huh…for them. It is a good thing. Uh huh….so we roll it over so that if everyone can get an opportunity to participate, it will help because the reputation of the nursing and midwifery profession is not a good one. And we are all ready to turn on a new leaf in the profession so that the bad reputation can be eliminated. So my main request would be if they can run (the workshops), because I know people who are coming there, apart from the mentoring we are giving them in the wards, till a new phase doing it, at the other times, then it will be fine. Yeah.

I: They will do them. Ma, when someone is coming here to deliver a baby, what is the cost?

R: It depends if it is SVD….

I: SVD or…

R: …and the suit within which she would be admitted; whether four in a room or one in a room. Those are the cheap suits that I have here.

I: OK.

R: Ahaaa….One in a room, SVD is thousand (1000GHC).

I: Oh OK.

R: For twenty-four (24) hours. And if it is CS, one in a room is….no, the CS for one in a room is three thousand, one hundred (3100GHC) for three (3) days.

I: Oh OK.

R: Including the day of admission, so it comes to second day post-op. But in my ward, there are sometimes clients here, third day, she is from far away, you see? So she will plead and sleep over the third day, and then we will discharge her. So they will make it up to four (4) days…

I: Oh OK.

R: ….so it becomes third day post-op. And if it is four in a room for CS, it is two thousand, seven hundred (2700GHC).

I: OK.

R: The SVDs, a thousand four (1400GHC) and eight fifty (850GHC) for four in a room.

I: OK.

R: That one is not even up to a thousand. And when you come, we sit you down and whichever your resources can match….there are people who use corporate insurance policies…

I: Oh OK.

R: There are some that we do accept; we accept Premier, we accept Cosmopolitan, we accet Acasoa….which one is a part of the group? I think they are about five (5) or so. So if you had any of these (insurance providers) and you come, we take you through the process and they will prepare your fist format for you and on the day you are discharged, you will go and complete the form and then you give it to them. And if they call us to let us know you are settled, we clear you for you to leave. But if you don’t have one of these…..as for health insurance (NHIS), we do not accept it.

I: You do not accept the local health insurance?

R: No, we do not accept that.

I: OK. I see. Ma, we are very grateful for the fact that you had this conversation with us this afternoon. We are most grateful.

R: No thanks needed.

VOICE 020. 23 MINUTES 56 SECONDS.

I: Good morning in-charge.

R: Good morning.

I: Please how are you doing?

R: By God’s grace I’m fine.

I: Okay. Do you’ve any question to ask about what we have already discussed?

R: There’s no question.

I: If there is no question can we continue our discussion?

R: Yes please.

I: Thank you very much. Please how old are you?

R: Thirty-three (33).

I: What is your educational qualification?

R: Tertiary.

I: What is your years of experience?

R: Four (4) years.

I: Please do you have a child?

R: Yes please.

I: Please how many are they?

R: Two (2).

I: Are you married please?

R: Yes please.

I: Thank you. We are discussing about respectful maternal care how do you understand it?

R: Respectful maternal care is about respecting and taking care of a mother to deliver safely

in a peaceful environment.

I: Please can you mention any keyword under respectful maternal care?

R: That is to respect the mother who is in to deliver.

I: Okay. Providing emotional support is to offer respectful and dignified care. As an in-

charge, what role do you play to promote respectful maternal care?

R: On duty I make sure every client is treated as expected, providing privacy and also

dusting because the client’s bed must be clean and well kept.

I: What strategies do you adopt to help women in labor whose actions direct or indirect

may put baby and themselves at risk or danger?

R: I try to calmly talk to such client and give her sacral massage if I’m the one taking care

of her or if I assigned someone, I make sure the person does same. We tell her the

implications of what she’s doing if she understands fine if she doesn’t we give her time

and later attends to her. At times we call doctors if required.

I: Have you ever apologize to a woman after helping her deliver safely with any of the

strategies you just mentioned? Maybe you shouted at her during delivery when she

wasn’t complying to instructions.

R: Personally I do, not even after delivery even in the course of delivery if I shout at you I

do apologize not intentional but have to do that for her to comply to instructions.

I: Have you received any report from a health provider for treating a woman roughly even

in our setting shouting or beating someone?

R: Report from a woman?

I: A mother. So have you receive a report from a healthcare provider that some of the

midwives here have treated a mother roughly when delivering them at the facility?

R: For here…(kiss teeth) no.

I: Maybe a patient can complain about a midwife who came on night duty or something.

R: Personally I’ve not encountered such. Sometimes a client behavior can make you voice

loud but I have not heard any making complains.

I: Have you seen or heard not what they will complain but maybe you’ve witnessed a

colleague shouting on a client?

R: Well, sometimes it does happen but when you sit to reflect on what would have

happened you see it as an African behavior some clients liked to be pampered but if you

don’t shout on them they will make delivery difficult.

I: As you’ve seen and heard can you describe how it actually happen in that situation?

R: It was just a normal delivery but the woman wasn’t cooperating. At the second stage, she

seems exhausted raising her leg here and there was difficult to calm her down so for the

midwife to offer a safe delivery she raised her voice and told the client if she doesn’t

push down the baby, she will stop what she’s doing and leave or beat her thigh though

she didn’t put that into action she just said that for the client to push down the baby.

I: In this particular instance, what was your response to your midwife?

R: Immediately I didn’t respond but afterwards I told her to be patience with the clients no

matter their behavior because as we are offering safe delivery we should make sure not

to lose our job.

I: What was the outcome after you spoke to the person?

R: Oh I’ve not seen her doing that anymore.

I: Have you ever seen or heard a healthcare provider treating a woman without seeking her

consent or not asking for permission when delivering at the facility?

R: Oh no.

I: Or someone complaining she didn’t know all she saw was being injected or calling her

husband that their baby will be injected with hepatitis b vaccine while the mother has

not even informed her husband?

R: Okay.

I: Your role as an in-charge promoting respectful maternal care we’re looking at privacy.

How many privacy screens do you have?

R: For now we’ve three (3) functioning privacy screens.

I: Are they adequate?

R: No because in a case we’ve three (3) clients then it means some won’t get a screen but

rather have to plead with the person and deliver her.

I: How do you ensure that your ward has adequate private screens to protect the privacy of

the child-bearing woman? In that we’re asking which unit in the hospital do you interact

with?

R: We write a request but you know this delays and others but we’re hoping they grant our

request.

I: So who do you interact with to get these screens?

R: Directly with our chief then we go to..

I: Chief Nursing Officer?

R: Yes Chief Nursing Officer then to the HOD and the business manager.

I: Okay. What challenges do you face in ensuring that your ward has adequate resources

and facilities to provide by officer? In that I want you to discuss your frustrations and

your responses to these challenges.

R: Well, sometimes looking at our cubicle you can deliver someone with head in vagina but

because there’s no screen to provide privacy you feel bad about why the request has

kept long but what can we do we’ve to improvise with her cloth so that we can provide

privacy.

I: We want to look at in-service training for new midwives and existing staff. As an in-

charge, erm do you take your staff through the right of a child-bearing mother?

R: Erm…like when they come new we try to do some in-service training for them though

not regular but we try doing it.

I: So if you take them through the training on client rights what are your impressions on

your staffs respond to that?

R: Erm…I didn’t know there’s unity even before I came here I realize what you will hear

outside about Komfo Anokye Labor ward is different inside. Whatever been thought here

we all try our best to put that in practice to get a good name.

I: Sometimes do your staff perceive it as monotonous as in calling a meeting and they’ll be

complaining that is same old story so we’re not coming?

R: Oh is not like that. They all participate.

I: Meaning every staff?

R: Every staff can’t be present some will be unavailable during the time is been held but

when it is held twice some will be present today the absentees will present another day

due to shift so some of our meetings are done on the Whatsapp platform.

I: Do you take your staff through how child-bearing women are respected and treated with

dignity?

R: Oh yes.

I: Okay. What challenges do you encounter in training your staff?

R: One of the challenges is what I just said because of our shift system is difficult to get all

staff to be present at a meeting. Apart from that we do well the staff always try their

best.

I: Working with the social welfare department of the hospital. One aspect of respectful

maternal care is trying to keep patient or mothers even after they have delivered because

they can not settle their hospital bill. What do you do when it is reported to you some

women cannot pay for their services?

R: For here it is hard to hear about such situations because after delivery, two (2) or three

(3) hours they’re sent to the lying ward it is there that they are discharged so if there’s

any challenges it will be there but for us no.

I: Okay. So that means you don’t resolve issues regarding discharging of your patients.

R: Oh no.

I: So in anyway do you work with the social welfare department?

R: Directly no.

I: Not directly. Measures to receive report of abuse from child-bearing mothers that is the

last thing we’re going to look at. Do you’ve any reporting system available to encourage

child-bearing mothers to report any abuse? When a patients makes the work complicated

and have no option but to tie her legs to the lithotomy bed what system is available for a

patient to report such situation?

R: We have no organized or specialized system but if someone is treated unfairly the person

can report to the Chief but we don’t really have a reporting system mainly for that…

I: That is your Chief Nursing Officer?

R: Yes. That this is where to report any abuse.

I: So have you ever receive report from child bearing mothers?

R: I have heard one but I don’t know much about it.

I: Was it reported to you?

R: No.

I: But you heard about it.

R: Yes I heard about it.

I: So what happened?

R: That it what I’m saying I can’t really remember I just heard and at the end the client

wasn’t cooperating and resulted in reporting.

I: Okay. With that particular instance what type of abuse was reported is that the shouting

erm..

R: Yes, yes shouting.

I: Physical restrain?

R: No. For physical restrain it has never happened even if it will happen it is only the

shouting maybe you didn’t talk well to a client.

I: Erm…if the systems are available how friendly will it be to all classes of women? With

the system of reporting you are saying there’s no specialized system however they’re

able to report it is very friendly to all categories of women such as adolescent girls,

educated, uneducated and people with disability?

R: It is not friendly I think if we’ve any specialized report system it will even help us to be

alert if we do something wrong and besides before someone will report to the chief

unless that person knows we’ve an officer you can report to else a lot doesn’t know. To

me I don’t see it as friendly to all manner of persons.

I: If there are systems in place to routinely monitor or investigate occurences of

mistreatment even in the absence of received report from…what we’re trying to find out

here is that are the systems in place to routinely monitor or investigate these occurences

of received reports from these women?

R: Erm… I think if it is well established it will help us so if the systems are put in place it

will be better. For us we’ve our incident that we report that it shouldn’t have happened

what about the client? So that is what we’ve to look at.

I: Is there anything you’ll like to bring up concerning what we’ve discussed?

R: I will say whatever we are doing if the outcome comes with lapses we can continue with

workshops so that we as midwives will work effectively and it will help us.

I: Okay please is there anything again?

R: No please.

I: Thank you very much for the interaction.

R: Thank you also.
